# Using latent variable models to make gaming-the-system detection robust to context variations

**DOI:** 10.1007/s11257-023-09362-1

**Published:** 2023-05-18

**Authors:** Yun Huang, Steven Dang, J. Elizabeth Richey, Pallavi Chhabra, Danielle R. Thomas, Michael W. Asher, Nikki G. Lobczowski, Elizabeth A. McLaughlin, Judith M. Harackiewicz, Vincent Aleven, Kenneth R. Koedinger

**Affiliations:** 1 Carnegie Mellon University, Pittsburgh, USA; 2 Lexia Learning, Concord, USA; 3 University of Pittsburgh, Pittsburgh, USA; 4 University of Wisconsin–Madison, Madison, USA

**Keywords:** Gaming the system, Gaming detector, Latent variable models, Item response theory, Behavior modeling

## Abstract

Gaming the system, a behavior in which learners exploit a system’s properties to make progress while avoiding learning, has frequently been shown to be associated with lower learning. However, when we applied a previously validated gaming detector across conditions in experiments with an algebra tutor, the detected gaming was not associated with reduced learning, challenging its validity in our study context. Our exploratory data analysis suggested that varying contextual factors across and within conditions contributed to this lack of association. We present a new approach, latent variable-based gaming detection (LV-GD), that controls for contextual factors and more robustly estimates student-level latent gaming tendencies. In LV-GD, a student is estimated as having a high gaming tendency if the student is detected to game more than the expected level of the population given the context. LV-GD applies a statistical model on top of an existing action-level gaming detector developed based on a typical human labeling process, without additional labeling effort. Across three datasets, we find that LV-GD consistently outperformed the original detector in validity measured by association between gaming and learning as well as reliability. LV-GD also afforded high practical utility: it more accurately revealed intervention effects on gaming, revealed a correlation between gaming and perceived competence in math and helped understand productive detected gaming behaviors. Our approach is not only useful for others wanting a cost-effective way to adapt a gaming detector to their context but is also generally applicable in creating robust behavioral measures.

## Introduction

Assessing students’ engagement levels or motivation from their interaction behaviors in digital learning environments is a compelling challenge both practically and theoretically. Practically, valid behavioral assessment of student engagement can drive adaptations that adjust to students’ needs, leading to greater learning and motivation; theoretically, it can be used to better understand when and why interventions or system designs work for enhancing student learning or motivation. One frequently explored behavioral indicator of student engagement is “gaming the system” (abbreviated as “gaming” in this paper), which is defined as “attempting to succeed in an educational environment by exploiting properties of the system rather than by learning the material and trying to use that knowledge to answer correctly” in the seminal works from Baker et al. (2006a, b, 2008a, b). Typical gaming-the-system behaviors include help abuse (e.g., copying the answer from a hint, repeated help requests) and systematic guessing (e.g., quickly answering after errors, making successive errors) (Paquette et al. 2014). Many studies have demonstrated that gaming the system is associated with poor learning outcomes in the short term or the long term (Almeda and Baker 2020; Baker, Corbett, Koedinger, and Wagner 2004; Cocea et al. 2009; Peters et al. 2018; San Pedro et al. 2013). Prior research suggests that interventions directly targeting gaming can reduce gaming behaviors (Baker, Corbett, Koedinger and Roll 2006a; Walonoski and Heffernan 2006b) and improve learning (Baker, Corbett, Koedinger and Roll 2006b), demonstrating the practical value of gaming detection. Recent work (Richey et al. 2021) has also shown that the positive effect of learning with an educational game was fully mediated by lower levels of gaming the system, showcasing the theoretical value of gaming detection for understanding how a specific intervention influences learning.

Over the course of development of gaming detectors, one problem that challenges the validity and practical effectiveness of gaming detectors has emerged but has not received enough attention: detected gaming is not always associated with poorer learning. In the seminal and subsequent works on gaming detectors, the avoidance of learning has been explicitly stated in the definitions of gaming (Baker et al. (2006a, b, 2008a, b; Cocea et al. 2009; Muldner et al. 2011). Thus, theoretically, the unproductiveness or harmfulness for learning is implied in the gaming construct.^1^ Practically, a gaming detector that only detects behaviors unproductive or harmful for learning also has higher effectiveness than a detector that does not have such a constraint. If a system intervenes when students are productively engaged due to false alarms of gaming, it may impair learning and reduce students’ trust in the system, leading to actual disengagement and greater learning impairment. Thus, the negative association between gaming measures and learning should be an important aspect of the validity of a gaming detector. However, this property is not inherently guaranteed in the operationalization of gaming, i.e., the detected gaming behaviors.

Our direct application of a previously validated gaming detector (Paquette et al. 2014) showed that detected gaming behaviors were associated with higher learning for a large proportion of students, and were not associated with learning for the overall population (reported later in this paper). Several prior works have also identified cases where detected gaming behaviors were not harmful for learning (Baker, Corbett and Koedinger 2004; Baker et al. 2008a, b; Cocea et al. 2009), or even appeared to be productive for learning (Shih et al. 2008). For example, in some cases the identified behavior of bypassing hints in search of bottom-out hints acting as worked examples can be viewed as “a positive meta-cognitive strategy that is only related to gaming the system at a surface level” (Baker et al. 2013, p. 16). Such productive detected gaming where gaming^2^ was associated with higher learning reduces the validity and practical effectiveness of the gaming detectors.

To address this, we only found one approach proposed in Baker et al. (2008a, b), where they used pretest and posttest scores to constrain the detected gaming labels to be assigned only to actions from students with low learning gains and low pretest scores. This approach utilizes the information from the “effects,” i.e., learning gains; we wonder whether we could utilize information from the “causes,” i.e., contextual factors that trigger productive detected gaming, which allows for generating more actionable insights than the former approach. Limited contextual factors have been considered during the human labeling process (in text replays or live observations), which usually sets the “ground truth” for developing gaming detectors (Baker, Corbett, Koedinger, and Wagner 2004; Baker and de Carvalho 2008; Walonoski and Heffernan 2006a). Typically, human coders make judgments of gaming based on observed information (e.g., action time, correctness, help request) from an individual student within a segment (e.g., five consecutive actions in text replays or 20 s in live observations). They do not interpret a student’s behavior in relation to the *population* behaviors on the task, i.e., the general propensity of a task to trigger detected gaming due to its design; nor do they consider a student’s knowledge on the task or the learning progression across tasks beyond the current segment. The limited contextual information used by human coders makes labeling faster and easier but risks introducing bias. For example, a student’s multiple fast, wrong attempts on drop-down menus in a segment may be labeled as gaming considering only her information within the segment, but if she did not deviate from the general behavior of the population on such steps and got similar steps correct in the future, then it may be more accurate to label these actions as not gaming.

To integrate contextual factors that may account for productive behaviors but are not considered in the original labeling processes, we propose *latent variable-based gaming detection* (LV-GD), an approach that integrates contextual factors in a cost-effective way for more valid and robust gaming assessment. The cost-effectiveness of LV-GD lies in applying a statistical model on top of an existing gaming detector developed based on a typical human labeling process, without additional labeling effort. The validity and robustness of LV-GD benefits from interpreting a student’s behaviors in relation to the population's behaviors in the same contexts represented by critical contextual factors. In the following subsections, we review related work, motivate the current work, and then introduce our approach and study in more depth.

### Contextual factors for detected gaming behaviors

In this section, we review two kinds of contextual factors that might trigger productive behaviors detected as gaming, but are not considered in typical human labeling processes. One kind is task features, i.e., characteristics of a problem, a set of problems (e.g., lessons, sections), or a system. Baker et al. (2009) examined a year-long log dataset with 22 different lessons of Cognitive Tutor Algebra and identified a set of task features that explained 56% of the variance in gaming, over five times the degree of variance explained in any prior study of student individual differences and gaming. For example, one such feature is “proportion of hints in each hint sequence that refer to abstract principles.” Results showed that gaming was more frequent in lessons that were abstract, ambiguous, and had unclear presentation of the content or task. Although the authors did not investigate whether detected gaming behaviors in such contexts were associated with better learning, results did suggest that in less well-designed tasks, students may game to acquire necessary information to perform the task. In another study, Baker (2007) found that lessons explained over three times as much of the variance in gaming as student individual differences did. In particular, 31% of lessons had average gaming frequencies higher than 20% with three lessons even reaching 40%. Paquette and Baker (2017) also found that differences in gaming behaviors were more strongly associated with the learning environments than with student populations. Although this indicates the important role of task features in explaining gaming, they have not been considered in typical human labeling processes.

Another kind of contextual factor is students’ knowledge levels on tasks. Roll et al. (2014) showed that on steps for which students have low prior knowledge, avoiding help and entering wrong answers repeatedly (which may be traditionally considered as systematic guessing, a form of gaming) is associated with better learning than seeking help. Shih et al. (2008) provided evidence that when students bypass hints to get bottom-out hints (traditionally considered as help abuse, a form of gaming), they are sometimes seeking worked examples. Dang and Koedinger (2019) also suggested that detected gaming can be a desirable adaptive learning behavior when students encounter challenges far beyond their abilities. However, students’ knowledge levels have not been considered in typical human labeling processes. Moreover, it has not been investigated whether these two contextual factors can have an interaction effect on detected gaming behaviors.

### Existing gaming detectors

Past research has developed two classes of gaming detectors: knowledge-engineering models and machine-learned models. In knowledge-engineered models, experts develop rational rules (sometimes called patterns) that can predict well human labels of gaming, and such rules are used to identify gaming behaviors (Muldner, et al. 2011; Paquette et al. 2014; Walonoski and Heffernan 2006b). In machine-learned models, a function between a set of features (e.g., correctness on a step) and human coded gaming labels is learned on a given dataset where only predictive features are maintained in the model, and the final model is used to identify gaming behaviors (Baker et al. 2008a, b; Pardos et al. 2014; Walonoski and Heffernan 2006a). In defining rules or features for the detectors, the emphasis has mainly been put on student features (Muldner, et al. 2011; Paquette et al. 2014; Pardos et al. 2014), such as how a student utilizes help (Aleven et al. 2006) or makes errors (Walonoski and Heffernan 2006a), or a student’s estimated knowledge on the related skill (Baker et al. 2008a, b). Task features have received less attention. We have only identified two machine-learned gaming detectors that incorporated task features such as interfaces (e.g., multiple-choice or textbox; Baker et al. 2008a, b; Walonoski and Heffernan 2006a). Meanwhile, in knowledge-engineered gaming detectors, task features typically are not considered, i.e., rules to identify gaming behaviors are usually described in a task type-independent way (Muldner, et al. 2011; Paquette et al. 2014).

Among existing gaming detectors, one stands out due to its superior performance in recent comprehensive evaluations in terms of generalizability, interpretability, and development cost in new contexts: the knowledge-engineered gaming detector (Paquette et al. 2014), which is referred to as KE-GD in this paper. KE-GD was developed by using cognitive task analysis to elicit knowledge about how experts code students as gaming or not in Cognitive Tutor Algebra (Koedinger and Corbett 2006). It consists of 13 patterns of students’ systematic guessing and help abuse behaviors. KE-GD represents the broad class of behavioral detectors that are built based on rational rules specified by experts. KE-GD has been initially validated by its acceptable predictive performance on human labeled gaming (Paquette et al. 2014). Recent comprehensive studies (Paquette and Baker 2019; Paquette et al. 2015) further compared KE-GD with two separately validated, representative gaming detectors across multiple datasets collected from different systems: a machine-learned model (Baker and de Carvalho 2008), and a hybrid model (Paquette et al. 2015) that combines both knowledge engineering and machine learning. The comparisons focused on predictive performance of human labels of gaming in held-out test sets in the original data and two new datasets collected from two other learning environments; the comparison also considered the interpretability of models. Results showed that KE-GD achieved greater generalizability to new datasets (or systems) and interpretability than the machine-learned model, and achieved comparable to slightly better generalizability and interpretability than the hybrid model. Although the initial cost in developing KE-GD was higher than that of the machine-learned model, it could be directly used in new datasets without further cost since actions that match any of the 13 patterns can be directly labeled as gaming. However, one may need to retrain the machine-learned or hybrid model, which needs a machine-learned model as input, given the much lower (and even unacceptable) predictive performance of the machine-learned model than KE-GD on new datasets. However, despite its proven advantages, the gaming patterns of KE-GD do not consider task features or students’ knowledge levels, and the association between its detected gaming and learning has not been examined in prior studies. This raises questions of the robustness of KE-GD on systems or datasets beyond the ones examined by the authors.

### Evaluation methods for gaming detectors

In past work, the standard procedure to evaluate a gaming detector is as follows (Baker et al. 2008a, b; Pardos et al. 2014; Walonoski and Heffernan 2006a): two or more human coders label gaming on student attempts or actions by classroom observations or text replays; if the inter-rater reliability is acceptable, these labels are used as the ground truth to develop detectors where a detector with better predictions of human labels are preferred. However, as mentioned earlier, human labels could contain bias (due to not considering task features or individuals’ knowledge) against which even a high inter-rater reliability cannot safeguard.

One evaluation method that addresses this concern is to examine the association between gaming estimates and learning, which is also intrinsically required by the standard definition of gaming (Baker et al. 2006a, b, 2008a, b). Some prior works have examined and found higher gaming levels to be associated with lower learning (Baker, Corbett, Koedinger, and Wagner 2004; Mogessie et al. 2020; Muldner, et al. 2011; Richey, et al. 2021), but others have not (Dang and Koedinger 2019; Paquette and Baker 2019; Paquette et al. 2015). Examining the relation with learning has not been generally considered as an integral part of evaluating gaming detectors. However, a gaming detector can be viewed as an instrument for assessing the gaming construct, and thus, *validity* is of great relevance. Validity provides “an overall evaluative judgment of the degree to which empirical evidence and theoretical rationales support the adequacy and appropriateness of interpretations and actions based on test scores or other modes of assessment” in the seminal work (Messick et al. 1995). In particular, the association between gaming and learning is closely related to external validity (i.e., does the test have convergent, discriminant, and predictive qualities?).

Besides validity, there are other desirable properties for a gaming detector that can be considered in the evaluation. *Reliability* measures the consistency (e.g., correlation) of results of an instrument over multiple samples. Different samples can be collected in various ways, such as across time or across subsets of items, which correspond to different types of reliability with distinct focuses (e.g., test–retest reliability, split-half reliability). Reliability does not imply validity, but it places a limit on the overall validity of an instrument that aims at measuring stable attributes or traits of people. An instrument with both high reliability and validity is usually desirable. To the best of our knowledge, only one prior study has examined and demonstrated the reliability of their proposed gaming detector (Muldner, et al. 2011) where they focused on correlations of gaming estimates from two buckets by a random split of problems or students.

*Generalizability* is also a desirable property of a gaming detector. It refers to how well a detector developed based on a sample can make predictions or estimations on a new sample, where a new sample can be from a different set of problems, a different student population, or a different system. It has some overlaps with reliability but emphasizes the performance on the new sample rather than the consistency between the estimates of the two samples. This aspect has been examined in various prior works (Baker et al. 2008a, b; Paquette et al. 2015).

### Latent variable models and the trait-like property of gaming

Obtaining a student-level gaming assessment is valuable for studying the relation between student-level attributes (e.g., motivation, learning gain) and gaming for understanding causes of disengagement or the effect on gaming of an intervention. Existing gaming detectors have focused on observed action-level gaming assessment, i.e., whether an action is part of a sequence of gaming behaviors, and the student-level gaming assessment is obtained by computing the proportion of gamed actions (i.e., observed gaming frequencies) or the average of predicted probabilities of gaming on actions for each student (Baker et al. 2008a, b; Paquette and Baker 2017; Razzaq et al. 2005). However, such direct aggregation may be prone to bias as illustrated earlier. Dang and Koedinger (2019) showed that observed gaming frequencies failed to correlate with motivation, while a statistical model that estimates a latent gaming tendency on a student level controlling for curricular sections yielded strong correlations between motivation and gaming. This is an example of a latent variable model, although the authors did not explicitly describe it as such. *Latent variable models* (LVMs) estimate values of latent theoretical variables (e.g., abilities, attitudes) based on observed response variables (e.g., task performance, survey ratings) through a statistical model that models the observed variables as a function of the latent variables. They have been widely used in knowledge modeling for estimating students’ abilities or knowledge levels (Desmarais and Baker 2012). One widely used LVM for ability assessment is *item response theory* (De Boeck and Wilson 2004). Item response theory models the observed correctness (responses) on each item (e.g., a problem step) of each student as a function of item difficulties and student abilities and thus provides an ability estimate for each student controlling for item difficulties. In essence, if a student performs better than the expected performance of the population on the items, the student is estimated as having a high ability; for two students with the same proportion correct over items, the one who can get harder items correct is estimated as having a higher ability than the one who only can get easier items correct. Ability estimates obtained in this way are more accurate than simply looking at the proportion correct over all items. Despite the prevalence of LVMs in knowledge modeling, their application in behavior modeling has been limited. Only a handful of papers have applied LVMs to estimate students’ affects or attitudes where latent variables were theoretical constructs measured from surveys such as cognitive appraisal (Sabourin, Mott, and Lester 2011) or attitudes toward learning (Arroyo and Woolf 2005).

To use LVMs in gaming detection, one requisite assumption is the existence of a trait-like property of gaming, for which some evidence has been accumulated. Baker et al. (2008a, b) and Dang and Koedinger (2019) both showed that gaming was associated with a range of survey measures that measured students’ motivational goals, beliefs, and dispositions at the beginning of the use of the system. Muldner et al. (2011) found that student factors explained a much higher proportion of variance than problems (50% vs. 19%) and were significantly more consistent than problems in gaming proportions across randomly split samples. Although some studies support the state-like property of gaming (see those cited in Sect. 1.1), they could not rule out the trait-like property of gaming by their analyses. Several studies suggest that gaming is a mixture of state and trait (Dang and Koedinger 2019; Muldner et al. 2011; Peters et al. 2018), or the domination of the state-like or trait-like property depends on the system design (Botelho et al. 2021). For example, Muldner et al. (2011) showed that a regression model with both students and problems as predictors explained more variance (61%) than students alone (50%) or problems alone (19%), and both predictors were significant. Thus, we can view the gaming construct at two interconnected levels: the latent student level, which corresponds to trait-like gaming tendencies, and the observed action level, which corresponds to state-like gaming behaviors affected by contextual factors and latent gaming tendencies. If we are interested in obtaining a student-level gaming measure for student-level analyses, we should consider extracting the trait-like component of gaming from behaviors across contexts, and LVMs offer an effective way to do so.

### The current study

In the current study, we demonstrate a new approach, latent variable-based gaming detection (LV-GD), that integrates contextual factors in a cost-effective way for more valid and robust gaming detection. We report our comprehensive evaluation and applications of LV-GD to support the validity, robustness, and usability of LV-GD. In addressing the gaps in existing research pointed out above, our work makes two main contributions. One contribution is a general cost-effective approach that can adapt an existing gaming (or other behavior) detector to a new context by integrating contextual factors not originally considered. Another contribution is the use of latent variable modeling in behavior assessment, showing the value of latent trait level assessment which is different from the dominant observed action level assessment.

LV-GD estimates a latent gaming tendency for each student controlling for the propensity of contexts to trigger detected gaming. Essentially, it makes student-level judgments about gaming by looking at a student’s behaviors across different contexts and in relation to the population-level behaviors in the same contexts. A student is estimated as having a high gaming tendency if the student is detected to game more than the expected levels of the population in the same contexts. LV-GD can do so by latent variable modeling (and generalized mixed effect modeling specifically) that predicts the action-level gaming judgements from an existing gaming detector given hypothesized latent factors that trigger detected gaming, without additional requirements on human labeling. From the fitted student random intercepts, we obtain gaming tendency estimates as the gaming measure. In the current study, LV-GD is used on top of the gaming detector KE-GD, a previously validated detector (Paquette and Baker 2019; Paquette et al. 2015); however, LV-GD can be used on top of any action-level gaming detectors.

Our study on LV-GD is reported in the following structure. Section 2 describes the development of LV-GD. We started with applying KE-GD on a dataset collected from experimentation with an algebra tutor. Observing the lack of association between detected gaming and learning, we conducted an iterative exploratory data analysis informed by prior research, and identified overlooked contextual factors. We then formulated LV-GD by incrementally integrating the contextual factors and exploring variants, chose the best model, and established initial validity. In Sect. 3, we examine the generalizability of LV-GD on new datasets in terms of generating gaming measures that can be negatively associated with learning. We compared LV-GD with KE-GD in nine contexts, obtained from three datasets with three condition configurations per dataset. To further support the validity of LV-GD, in Sect. 4 we examine reliability of LV-GD in comparison with KE-GD, which can also be viewed as further evaluating generalizability: how well does a gaming detector generalize to new contexts in terms of having consistent latent gaming tendencies? In Sect. 5, we demonstrate three applications of LV-GD: to study intervention effects on gaming, to explore the relation between gaming and motivation, and to help understand productive detected gaming behaviors through a qualitative analysis. Finally, in Sect. 6 we conclude and discuss the results.

## Development of LV-GD

### The tutor

**Fig. 1:**
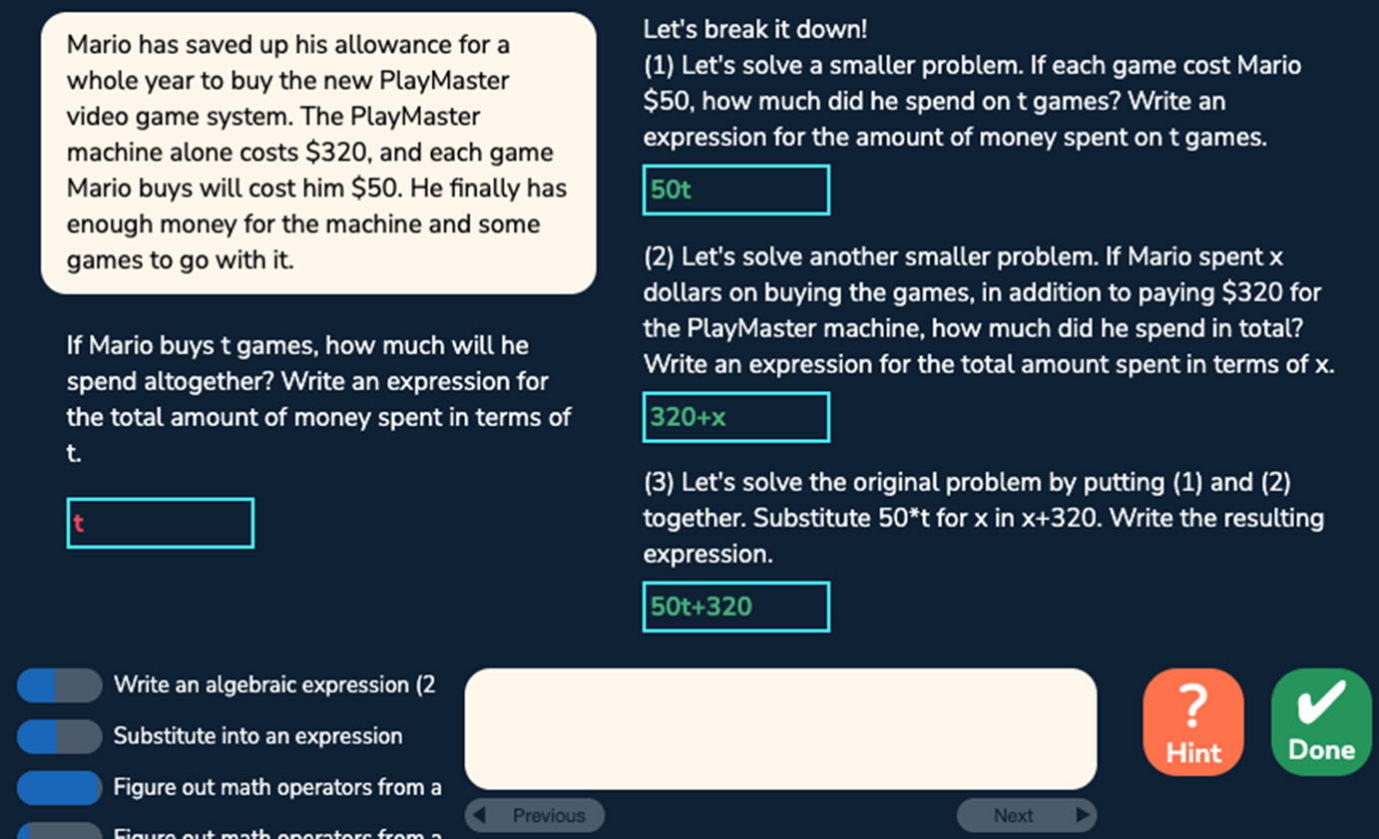
A problem with the text format

**Fig. 2:**
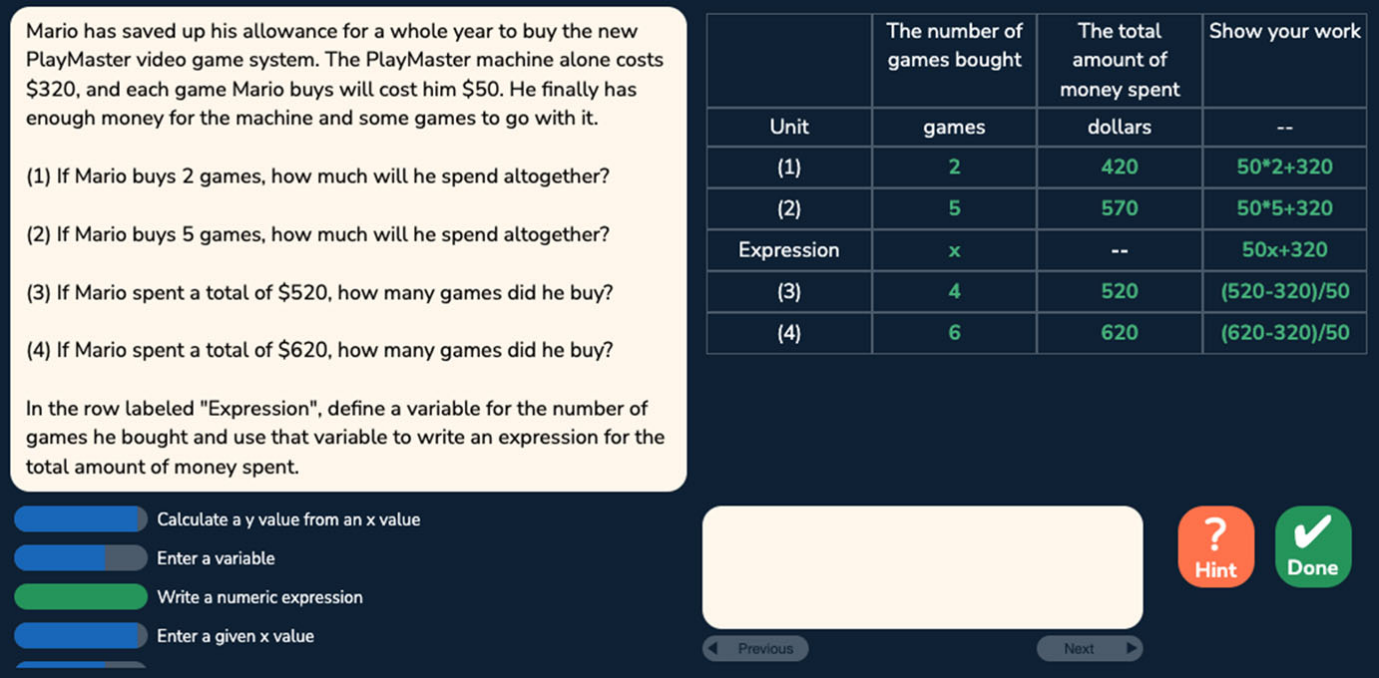
A problem with the table format

**Fig. 3:**
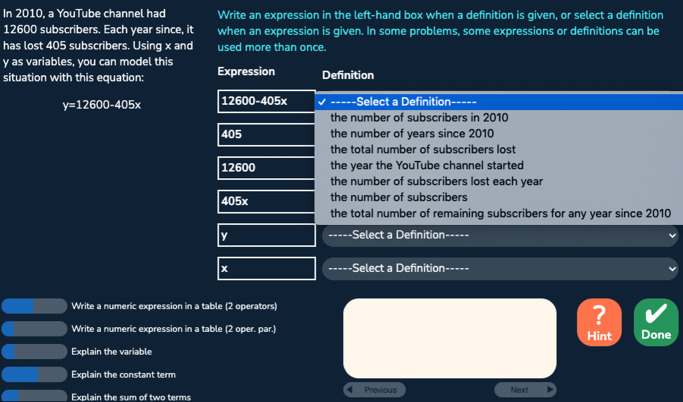
A problem with the menu format

**Fig. 4:**
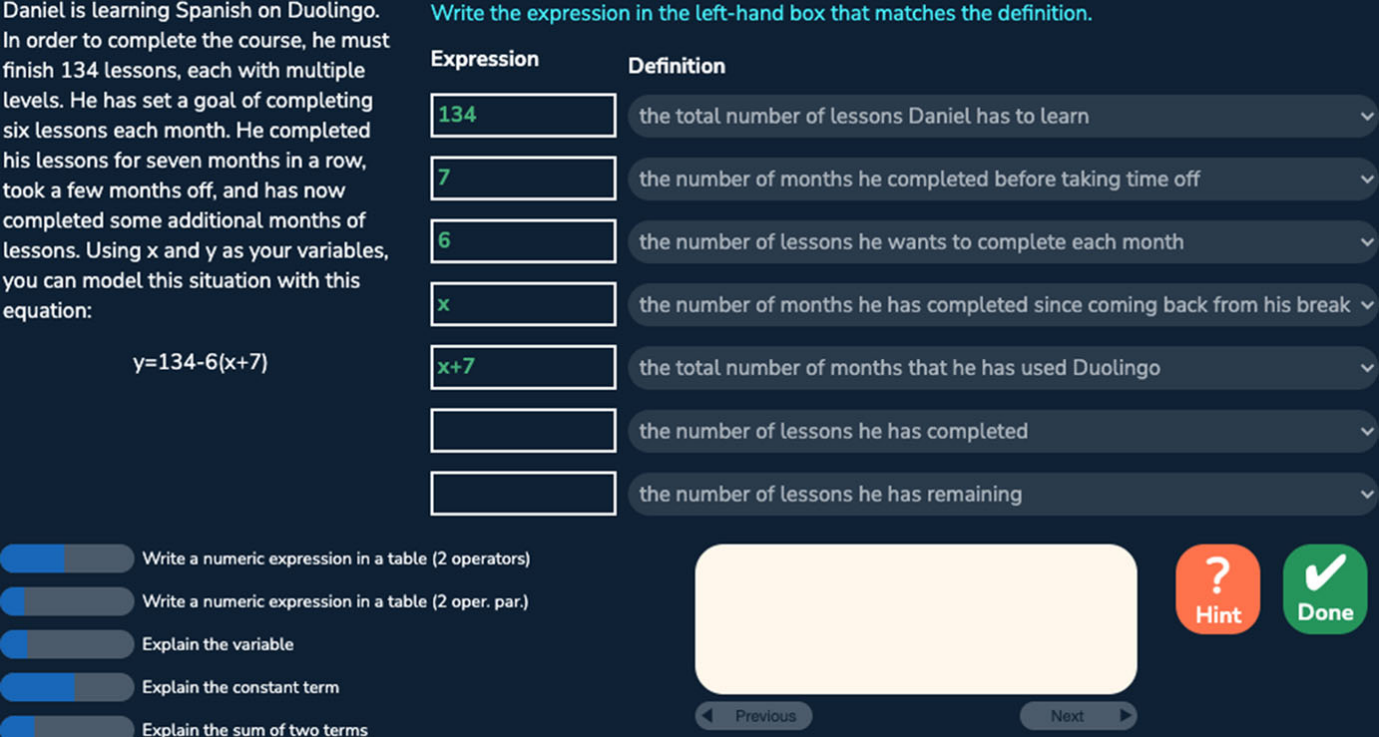
A problem with the flipped-menu format

We used datasets collected from an algebra intelligent tutoring system for middle and high school students (Huang et al. 2021). Students learn about writing algebraic expressions in story problems in various formats: writing an expression in a textbox with dynamic scaffolding steps that appear if a student fails in the original question (*text* format as shown in Fig. 1); writing expressions in a table where the main question step and scaffolding steps are accessible at any time and are all required (*table* format as shown in Fig. 2); explaining a set of expressions extracted from a given equation by choosing the matching textual description from a dropdown menu for each expression (*menu* format as shown in Fig. 3); and given an equation, writing a set of expressions that match a given set of textual descriptions (*flipped-menu* format as shown in Fig. 4). These tasks also vary in the complexity of the expressions involved (e.g., one or two operators).

The algebra tutor was continuously redesigned and tested in three experiments with different student populations across 3 years. In each experiment (eight sessions over 4 weeks), we compared two versions of the tutor corresponding to two conditions differing in task design and sequencing. The control (CT) condition, corresponding to the original tutor, provided a *normal deliberate practice* schedule, where students received tasks with feedback and as-needed repetition for improving critical aspects of performance. Students received full tasks representing the full version of the problem and were required to fill in all steps (including scaffolding steps) given a cover story. There were three consecutive units: the first unit contained all the table tasks, the second unit contained less complex menu and flipped-menu tasks, and the third unit contained more complex menu and flipped-menu tasks. Steps were labeled with coarser-grained knowledge components (KCs; skills). Students received individualized practice until reaching mastery of all KCs in a unit before moving on to the next unit. Across the three experiments, the design of the control condition remained the same. The experimental (EXP) condition corresponds to a redesigned tutor based on data mining outcomes such as a refined KC model revealing hidden difficulties after original KCs were split to differentiate easier and harder use cases. It provided an *intense deliberate practice* schedule where students practiced on a larger number of KCs with a higher variety of tasks targeting different subsets of KCs. Focused tasks were introduced to reduce over-practicing easier KCs and target particularly difficult KCs. Examples of focused tasks include: text format tasks asking for the final expression without the mandatory intermediate steps required in the table task; text format tasks that further remove the story and focus on learning algebraic grammar rules; and simpler menu and flipped-menu tasks with equations less complex than the original equations. There were three or more learning units where different task formats or task types (full or focused) were interleaved in each unit. Students received individualized practice until reaching mastery of all KCs in a unit before moving on to the next unit. Across the three experiments, the design of the experimental condition was continuously refined to promote greater learning. Our prior work has shown that the experimental condition led to better learning outcomes compared to the control condition (Huang et al. 2021). Here, we are interested to see whether the experimental condition also led to higher behavioral engagement, particularly lower levels of gaming the system, and whether gaming was linked with motivation. We started our investigation with the first dataset collected from the first experiment explained below.

### A previously validated gaming detector did not generalize

We chose a previously validated knowledge-engineered gaming detector, KE-GD, as the starting point for studying students’ behavioral engagement when using the algebra tutor. KE-GD contains 13 interpretable patterns modeling systematic guessing and help abuse. For example, one pattern is “the student enters an incorrect answer, enters a similar and incorrect answer in the same part of the problem and then enters another similar answer in the same part of the problem.” It is coded as “incorrect → [similar answer] [same context] & incorrect → [similar answer] & [same context] & attempt,” consisting of constituents such as “[similar answer]” (judged by Levenshtein distance), and action types such as “attempt” (correct or incorrect) or “help.” If a sequence of actions (i.e., attempts on steps) matches any one of the 13 patterns, then all actions involved are labeled as gaming. Details of the patterns and the validation of KE-GD could be found in (Paquette and Baker 2019; Paquette, de Carvalho, and Baker, 2014).

We used KE-GD to label actions as gaming or not and then examined its validity. We defined two metrics of validity in the current study, both of which evaluate the association between gaming and learning. The primary metric was the correlation between gaming levels and normalized learning gains over students. For each student, we computed a *gaming level* using the proportion of gamed actions (referred to as *proportion of detected gaming* or *detected gaming (proportion)*) for KE-GD, or the estimated gaming tendency for LV-GD (explained in Sect. 2.3.2); we computed the normalized learning gain using the widely adopted formula, *(posttest—pretest) / (1- pretest).* We used Spearman correlation (*rho*) because it is less sensitive to outliers than Pearson correlation. As a supplementary metric, we conducted a regression analysis predicting posttest scores controlling for pretest scores and gaming levels over students and examined the coefficient of the variable of gaming levels. We considered negative correlations and coefficient values at a significance level of 0.10 as acceptable validity. Prior studies have used significance levels of 0.05 and 0.10 for correlation analyses involving behavior measures (Baker et al. 2004a, b; Dang and Koedinger 2019; Shih et al. 2008).

Two observations emerged. First, the detected gaming proportion 18% (last column in Table 1) was much higher than the previously reported proportions (3.5% in Dang and Koedinger (2019) and 6.8% in Paquette et al. (2014)) of the same detector in other math intelligent tutoring systems. Second, there was a lack of association between detected gaming and learning (correlation: *rho* =  − 0.02, *p* = 0.86; regression coefficient: *b* = 0.07, *p* = 0.69), challenging KE-GD’s validity in our context.

**Table 1:** Statistics of the Fall 2019 dataset including gaming levels detected by KE-GD

#Stu	#Actions	#Actions of only 1st attempts of steps w/KCs	Avg proportion of gamed actions (considering all attempts of all steps) over students
129	98,176	32,419	.18 (*SD* = .08)

### Identifying and integrating contextual factors to improve validity

Next, we conducted iterative exploratory data analysis on the first dataset to identify contextual factors that might explain the lack of association between detected gaming by KE-GD and learning, and integrated the contextual factors through latent variable modeling analogous to item response theory modeling, explained as follows.

#### Identifying the effect of task formats

**Fig. 5:**
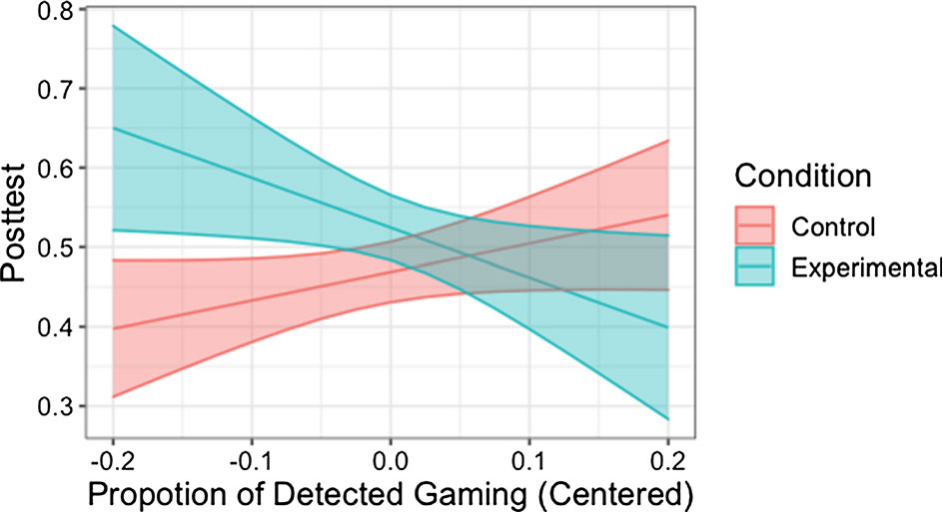
The interaction plot between the condition and detected gaming proportion of the regression model predicting posttest scores with pretest scores controlled for

One notable feature of our dataset is that it was collected from experimentation with two conditions with substantial differences in task design and sequencing. The experimental conditions constituted the largest contextual variations. So, we first conducted a moderation analysis to test whether detected gaming was associated with learning differently between the conditions. We constructed a regression model predicting posttest scores for each student given the pretest scores, the condition indicator, detected gaming proportion and an interaction term between the condition and detected gaming proportion. We found a significant, crossover interaction (*b* =  − 0.98, *p* = 0.007) where the control condition showed a relation opposite to theoretical prediction: higher proportion of detected gaming was associated with higher posttest scores (Fig. 5).

**Fig. 6:**
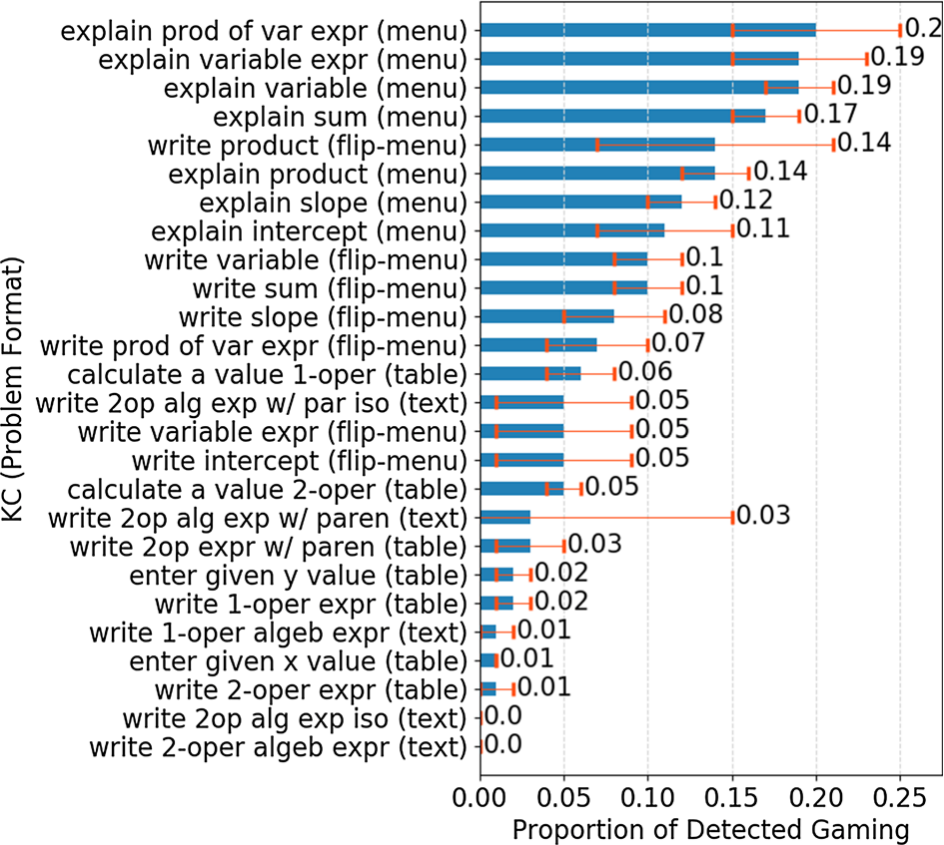
Detected gaming proportion by KCs averaged over students (95% confidence intervals are plotted; only first attempts of steps with KCs are considered)

To explain this, a natural hypothesis was that in some contexts detected gaming behaviors were productive learning behaviors. To identify such contexts, we broke down the largest context—experimental condition—into smaller contexts, to examine *where* detected gaming was particularly high on the overall dataset, which might suggest misclassification of productive behaviors as gaming. We used the unit of analysis normally used for modeling student learning, knowledge components (KCs), for better drawing insights into the relation between gaming and learning. We used the KC model previously validated for this dataset (Huang et al. 2021). It includes 26 KCs shared by both conditions.^3^ We first examined whether students gamed much more on some KCs than on others, and if so whether there was a pattern in this variation. A pattern emerged (see Fig. 6): KCs required in menu and flipped-menu formats had particularly high detected gaming levels, ranging from 5 to 20% on average, much higher than those required in table and text formats, In particular, the proportion on menu formats, 11% to 20% on average, was much higher than previously reported proportions (Dang and Koedinger 2019; Paquette et al. 2014). It is likely that some of the detected gaming behaviors on menu and flipped-menu formats were normative, productive learning behaviors.

**Fig. 7:**
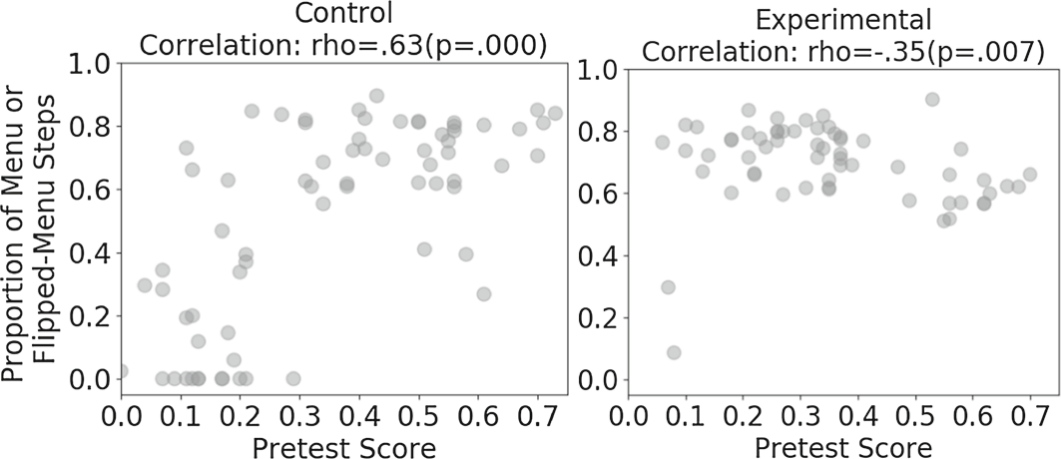
Correlations between pretest scores and proportion of highly gamed formats (menu, flipped-menu) per condition

Connecting the above finding with the previously discovered interaction, a natural next question was: did formats distribute differently in each condition? We examined the unit organization and found a dramatic difference: menu and flip-menu problems were positioned later in the control condition but were given across the units in the experimental condition. Having in mind that higher detected gaming was associated with higher posttest scores in the control condition (Fig. 5), we wondered whether this association was because students with higher abilities (who usually also have higher posttest scores) progressed faster to later units and thus accessed a higher proportion of menu and flipped-menu steps, which were highly gamed contexts, than students with lower abilities. To investigate this, we approximated students’ abilities by pretest scores and studied the correlation between pretest scores and proportion of menu and flipped-menu steps. Indeed, as shown in Fig. 7, students with higher pretest scores in the control condition accessed a higher proportion of menu and flipped-menu steps than students with lower pretest scores, which was the opposite to the experimental condition. Thus, the positive association between detected gaming and posttest scores in the control condition was due to a confounder, the proportion of highly gamed format steps a student accessed, which was itself confounded with student ability. A higher proportion of menu and flipped-menu steps was associated with a higher overall proportion of detected gaming; at the same time, it was also associated with higher posttest scores. Thus, this resulted in a spurious, biased relation where a higher proportion of detected gaming was associated with higher posttest scores. If we introduce task formats to account for the detected gaming, then this bias may be reduced.

#### The basic latent variable model controlling for task formats

Based on the above exploratory data analysis, we formulated a basic latent variable model, the simplest form of LV-GD, that explains detected gaming by both students’ latent gaming tendencies and formats’ propensities to trigger detected gaming. This is analogous to explaining item performance by both students’ latent abilities and item difficulties in the Rasch model (De Boeck and Wilson 2004), the simplest form of item response theory models. To illustrate our model, we would label a student with a high detected gaming level simply due to having a high proportion of the menu format as not actually having a high gaming level. We use a generalized linear mixed model that predicts the binary detected gaming label per action asserted by KE-GD, given the student identity (modeled as a random factor) and the format (modeled as a fixed factor):

1 $${\text{Detected}}\;{\text{gaming}}:\;{\text{G}}\sim \left( {{1}|{\text{Student}}} \right) + {\text{Format}}$$

2 $${\text{Gaming tendency}}:\; \alpha = {\text{exp}}(\theta)$$

Formula (1) is written using the syntax of R’s lme4 package for better replicability; a formal mathematical description is that the log odds of an action being labeled as gaming by KE-GD is a linear function of the student’s identity (of which the coefficient is the student’s random intercept $\theta$) and the current format. In formula (2), a student’s *gaming tendency* $\alpha$ is obtained by exponentiating the student’s random intercept $\theta$ from formula (1), converting log odds scale to odds scale. This basic model improved over KE-GD in terms of the sign and strength of the association with learning (rho =  − 0.07, *p* = 0.44; see Table 2 row #1); there was no longer an interaction between the condition and gaming levels (*b* =  − 0.04, *p* = 0.24). However, the association between gaming and learning was not statistically significant, demanding further investigation.

**Table 2:** Associations between gaming tendencies from variants of LV-GD and learning

ID	Level	Predictors	Cor with NLG	Post ~ Pre + G
1	Format	(1|Stu) + F	*rho* = − .07, *p* = .44	*b* = − .02, *p* = .31
2	Format	(1|Stu) + F + Pre	*rho* = − .14, *p* = .11	*b* = − 0.03, *p* = .16
3	Format	(1|Stu) + F + Pre + Pre:F	***rho* = − .16, *p* = .07**	*b* = − 0.03, *p* = .14
4	Format	(1|Stu) + F + Pre + Pre:F + Opp	***rho* = −.16*, p* = .07**	*b* = − 0.03, *p* = .14
**5**	**Format**	**(1|Stu) + F + Pre + Pre:F + Opp + F:Opp (The final chosen model)**	*rho* = − .18, *p* = .04*	***b* = − 0.04, *p* = .09**
6	Format	(1|Stu) + F + Pre + Pre:F + Opp + F:Opp (fitted on 1st attempts of steps w/ KCs)	***rho*** = − .26, *p* = .00**	*b* = − 0.08, *p* = .01*
7	KC	(1|Stu) + (1 + Pre + Opp|KC) + Pre + Opp (fitted on 1st attempts of steps w/ KCs)	*rho* = − .25, *p* = .00**	*b* = − 0.07, *p* = .01*
8	Problem	(1|Stu) + (1 + Pre|Problem) + Pre	*rho* = − .18, *p* = .04*	*b* = − 0.05, *p* = .04*

Correlations with normalized learning gains and coefficients of gaming tendency variables in regression predicting posttest scores are reported (*p* < .10: boldface *p* < .05: boldface and starred; F: Format, Pre: Pretest, Opp: Opportunity). Row #7-#8: KCs and problems had a high number of levels and were treated as random factors

#### Identifying other contextual factors

**Fig. 8:**
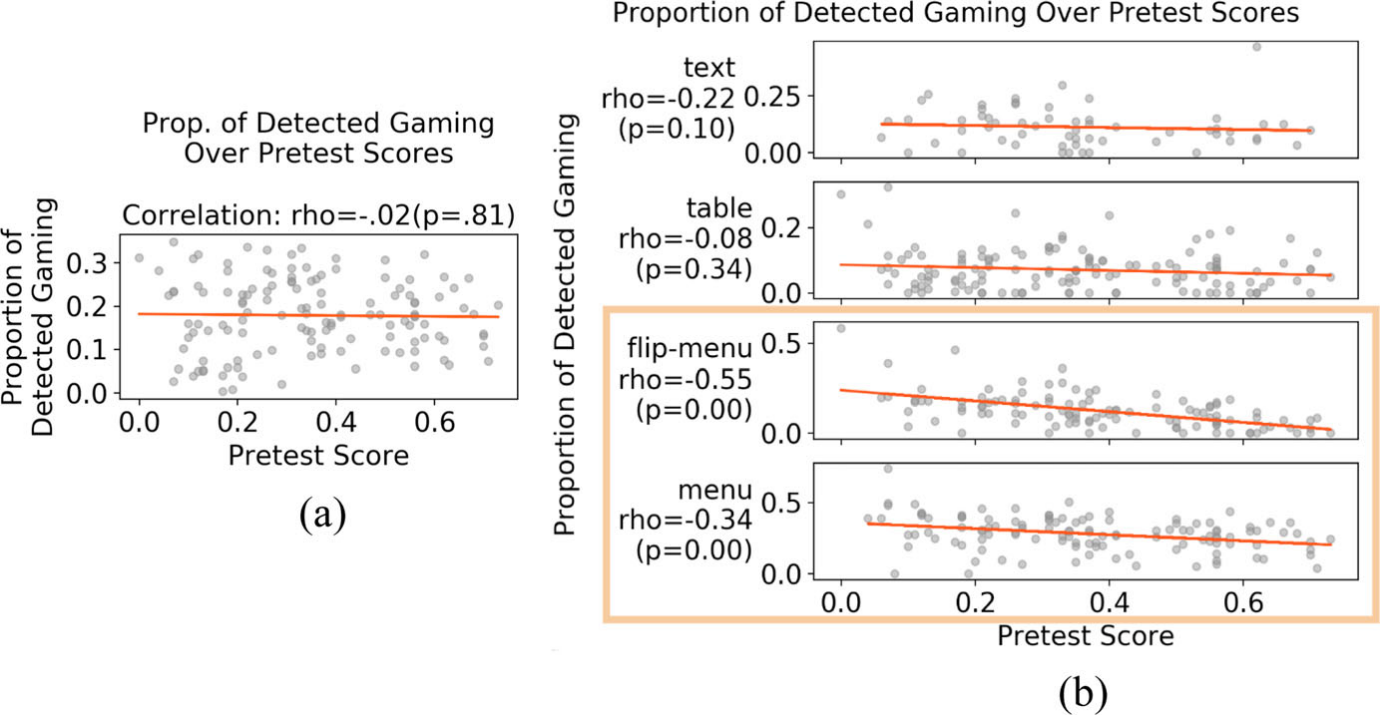
Correlations between pretest scores and detected gaming proportion overall and per task format over students (considering all attempts of all steps)

**Fig. 9:**
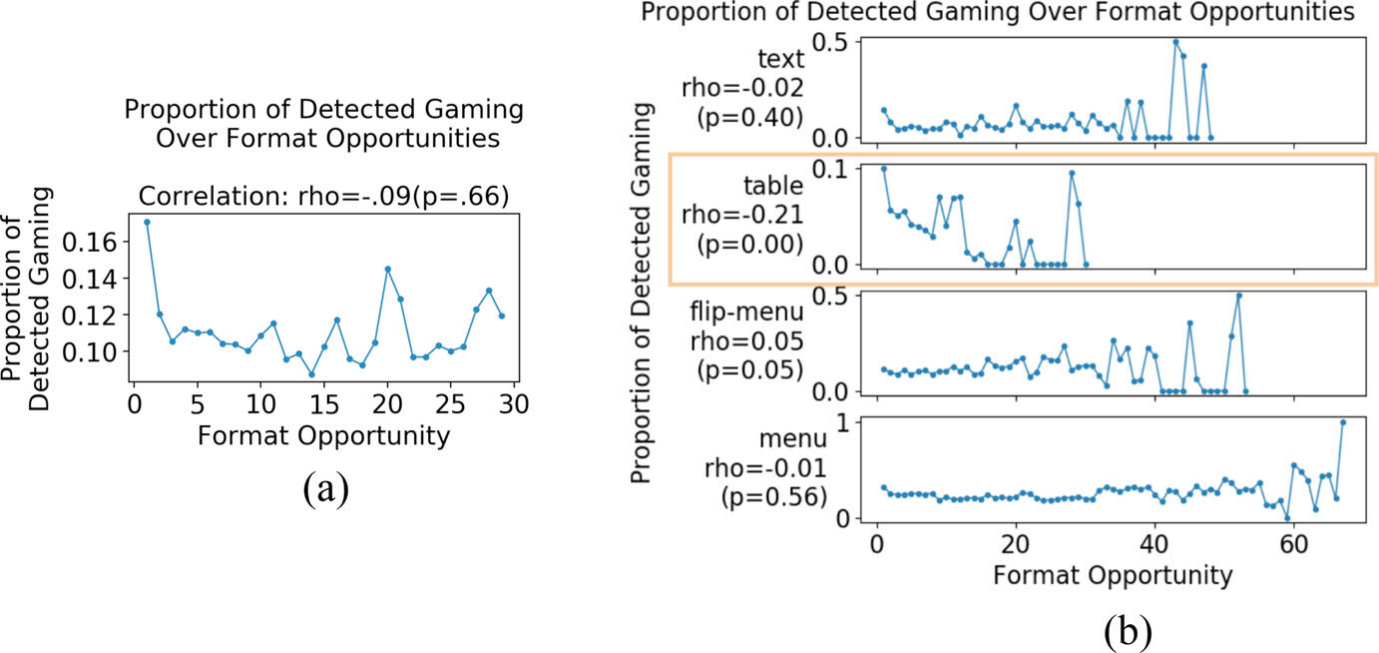
Correlations between practice opportunities and detected gaming overall and per task format. Each point corresponds to the average proportion of detected gaming at an opportunity over students (considering all attempts of all steps). All actions in the same problem have the same opportunity count for the corresponding format. The blips at the end of the curves are due to small sample sizes

We further examined *when* detected gaming was particularly high on the overall dataset, which might also suggest misclassification of productive behaviors as gaming. Based on literature review in Sect. 1.1, we hypothesized that students’ prior knowledge levels (approximated by pretest scores) and current knowledge levels (approximated by practice opportunities) are also contextual factors accounting for productive detected gaming: when students have lower pretest scores, they are more likely to try to learn by behaving in ways being classified as gaming than those with higher pretest scores. Likewise, when students are at earlier practice opportunities, they are more likely to try to learn by behaving in ways classified as gaming than at later practice opportunities. We conducted correlation analyses between detected gaming levels and students’ pretest scores or practice opportunities. Since we had already identified task formats as a contextual factor, we examined the correlations both overall and by format. Figure 8 shows that on flipped-menu and menu formats, students with lower pretest scores gamed much more than students with higher pretest scores, whereas this was not the case for other formats and overall. Figure 9 shows that on table formats, students were more likely to game on earlier than later opportunities and reduced gaming quickly over opportunities, whereas this was not the case for other formats and overall. Discussion of these findings can be found later in Sect. 6.2. Based on this analysis, we integrated the discovered contextual factors into a latent variable model explained below.

#### The full latent variable model accounting for critical contextual factors

From our two sets of exploratory data analyses, we obtained a key insight: different contexts have different propensities to trigger detected gaming under a given system design. A gaming detector should consider contexts’ gaming propensities in addition to students’ gaming tendencies. If a student gamed more than the expected detected gaming levels of the population in the contexts, then and only then should the student be considered as gaming. This is because the proportion of detected gaming is usually a low proportion of a population or a dataset (typically less than 7% in past studies and less than 20% in our datasets), so the detected gaming level of all students in a context represents the *normative behavior* of the population in that context. A model that describes well detected gaming behaviors of a dataset captures the normative behaviors of a population in the corresponding system, and the degree of *deviation* from the normative behaviors should represent the intended gaming construct. The full formulation of our latent variable model LV-GD is as follows (using a generalized linear mixed model):

3 $$\begin{gathered} \quad {\text{Detected gaming}}:{\text{ G}}\sim \left( {{1}|{\text{Student}}} \right) + {\text{Format}} \hfill \\ \quad + {\text{ Pretest}} + {\text{Pretest}}:{\text{Format}} \hfill \\ \quad + \;{\text{Opportunity}} + {\text{ Opportunity}}:{\text{Format}} \hfill \\ \end{gathered}$$

4 $${\text{Gaming}}\;{\text{tendency}}:\alpha = {\text{exp}}(\theta)$$

Formula (3) is written using the syntax of R’s lme4 package for better replicability; a formal mathematical description is that the log odds of an action being labeled as gaming (vs. not gaming) by KE-GD is a linear function of the student’s identity (of which the coefficient is the student’s random intercept $\theta$), the current format, the student’s pretest score, the interaction between the pretest score and the format, the practice opportunity count of a format of the student (note that all steps of a problem are considered as having the same opportunity count of the corresponding format), and the interaction between the opportunity count and the format. Except for the student identity modeled as a random factor, all other predictors are modeled as fixed factors. In formula (4), a student’s gaming tendency $\alpha$ in odds scale is obtained by exponentiating $\theta$ from formula (3).

**Fig. 10:**
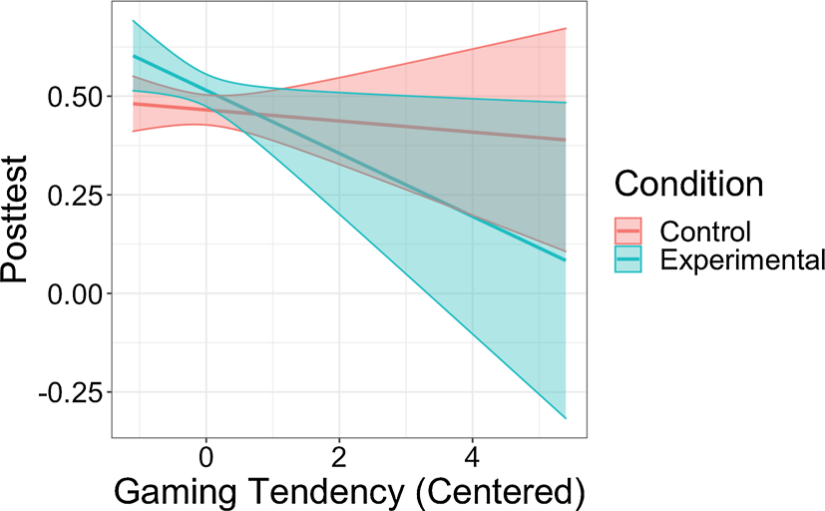
The interaction plot between the condition and gaming tendencies from the chosen model of LV-GD of the regression model predicting posttest scores

Table 2 shows the validity metrics of full models (row #5-#8) as well as reduced models (row #1-#4) of LV-GD. All the seven variants reached higher validity than KE-GD in terms of having stronger associations with learning, and the four full models reached desirable statistical significance (row #5-#8). The five predictors increasingly strengthened the association (except when adding the single opportunity term in row #4 before adding the interaction term) and were necessary for reaching acceptable validity in this dataset. In formulating the full models, we explored three other variants different from the one in row #6: one that used KCs as the unit (row #7) and fit the model using first attempts of steps with KC labels (without modifying the detected gaming labels associated with these actions); another that used the same data subset as the KC-level model to fit the model but maintained the unit of format (row #6); another that used problems as the unit (row #8) and fit the model with all attempts of all steps as the format-level model in row #5. We found that a format-level modeling worked as well as the KC-level modeling, when using the same subset (row #6 vs. #7); using the subset with only first attempts of steps labeled with KCs could improve validity compared to using all attempts of all steps (row #6 vs. #5) in this dataset; a problem-level modeling using problem labels from the data worked as well as a format-level modeling (row #8 vs. #5). We chose to fit LV-GD with all attempts of all steps using formats as the unit rather than using KCs as the unit or using only first attempts of steps with KC labels for potentially greater generalizability, since it does not require additional KC labels; we chose formats rather than problems as the unit due to coherence with findings from our exploratory data analysis and potentially greater generalizability to new problems. The final chosen model for the rest of the paper was the one in row #5 in Table 2. Under this model, there was no interaction between the condition and gaming levels (*b* =  − 0.07, *p* = 0.15); both conditions exhibited negative associations between gaming levels and posttest scores (Fig. 10). Its fitted parameters (Table 3) had high consistency with the patterns observed in exploratory data analyses; some differences may be due to the differences in statistical methods and data processing used in the two kinds of analyses. We thus concluded the formulation of LV-GD for valid gaming detection in our tutor.

**Table 3:** Parameters of the chosen full model of LV-GD (row #5 in Table 2)

Modeling purpose	Regression term	Coefficient
Effect of format	Intercept (Text)	$\beta$ = − 2.09, *p* < .001 ***
	Table	$\beta$ = − 1.50, *p* < .001 ***
	FlipMenu	$\beta$ = 0.09, *p* = .03 *
	Menu	$\beta$ = 1.12, *p* < .001 ***
Effect of prior knowledge adjusted by formats	Pretest (Pretest:Text)	$\beta$ = − 0.09, *p* = .13
	Pretest:Table	$\beta$ = 0.04, *p* = .37
	Pretest:FlipMenu	$\beta$ = − 0.19, *p* < .001 ***
	Pretest:Menu	$\beta$ = − 0.10, *p* = .01 *
Effect of current knowledge adjusted by formats	Opp (Opp:Text)	$\beta$ = 0.01, *p* = .71
	Opp:Table	$\beta$ = − 1.13, *p* < .001 ***
	Opp:FlipMenu	$\beta$ = − 0.00, *p* = .99
	Opp:Menu	$\beta$ = − 0.01, *p* = .84

Categorical variables were dummy coded and continuous variables were standardized for reducing multicollinearity. The coefficients are in log odds scale

## Generalizability of LV-GD

In the previous section, we conducted exploratory data analysis and validity evaluation on a single dataset, which might risk overfitting to the dataset. In this section, we tested the generalizability of LV-GD to two new datasets. More specifically, we examined whether the model structure (i.e., predictors and dependent variables) of LV-GD established from the first dataset can be generalized to new datasets for generating gaming measures that associate with lower learning. We looked into conditions separately and together for all three datasets, resulting in nine contexts across different student populations and designs of the system. The two new datasets were collected in 2020 Spring (20S) and 2021 Fall (21F) from the second and third experiments with the tutor in different schools, where some design changes derived from data mining were introduced in the experimental (EXP) condition: new units were introduced for providing focused practice on prerequisite KCs; a lower proportion of menu and flipped-menu tasks was positioned in earlier units compared to the first dataset; a new task format was introduced in the 21F dataset involving interactions with animations. The four task formats identified in the first dataset were still present in the two new datasets. On the other hand, the control condition remained the same.

Table 4 shows statistics of all datasets including detected gaming by KE-GD. Again, the detected gaming proportions were high (16%) in the new datasets. When applying both detectors to the nine contexts (see Table 5), LV-GD consistently outperformed KE-GD in reaching higher associations with learning in eight of the nine contexts. The exception was the EXP condition in the 21F dataset, in which the correlation of LV-GD was slightly weaker but of the same level of significance as KE-GD. In particular, when examining both conditions together and the EXP condition alone, LV-GD reached high validity (i.e., *rho* < 0 and *p* < 0.05) in all six contexts, while KE-GD only reached validity in half of the contexts. When examining the control (CT) condition, LV-GD also improved on KE-GD by reversing positive correlations to the theoretically consistent negative correlations for all three CT datasets and reached acceptable significance on the 20S CT dataset, although the correlations did not reach acceptable significance in other CT datasets. We conducted further investigation next.

**Table 4:** Statistics of datasets (CT: control condition, EXP: experimental condition)

Data	#students			#actions	Avg proportion of gamed actions over students (by KE-GD)
	All	CT	EXP		
19F	129	69	60	98,176	.18 (*SD* = .08)
20S	222	106	116	109,193	.16 (*SD* = .11)
21F	99	46	53	59,703	.16 (*SD* = .11)

**Table 5:** Associations between gaming from KE-GD or LV-GD with learning across nine contexts

Data	Detector	All		CT		EXP	
		Cor w/ NLG	Post ~ Pre + G	Cor w/ NLG	Post ~ Pre + G	Cor w/ NLG	Post ~ Pre + G
19F	KE-GD	***rho*** = − .02, *p* = .86	*b* = 0.07, *p* = .69	***rho*** = .14, *p* = .25	*b* = 0.34, *p* = .11	*rho* = − .29, *p* = .02*	*b* = − 0.71, *p* = .02*
	LV-GD	*rho* = − .18, *p* = .04*	***b* = − 0.04*, p* = .09**	***rho*** = − .02, *p* = .86	*b* = − 0.01, *p* = .62	*rho* = − .41, *p* = .00**	*b* = − 0.10, *p* = .02*
20S	KE-GD	***rho*** = − .04, *p* = .55	*b* = 0.01, *p* = .92	***rho*** = .16, *p* = .10	*b* = 0.25, *p* = .10	***rho*** = − .13, *p* = .17	*b* = − 0.32, *p* = .12
	LV-GD	*rho* = − .20, *p* = .00**	*b* = − 0.04, *p* = .00**	***rho* = −.19*, p* = .05**	*b* = − 0.05, *p* = .03*	*rho* = − .21, *p* = .02*	***b* = −0.03*, p* = .06**
21F	KE-GD	*rho* = − .29, *p* = .00**	*b* = − 0.45, *p* = .00**	***rho*** = .00, *p* = .98	*b* = − 0.04, *p* = .85	*rho* = − .52, *p* = .00**	*b* = − 0.83, *p* = .00**
	LV-GD	*rho* = − .36, *p* = .00**	*b* = − 0.05, *p* = .00**	***rho*** = − .20, *p* = .18	*b* = − .02, *p* = .19	*rho* = − .48, *p* = .00**	*b* = − 0.09, *p* = .00**

The Gaming variables in regression models predicting posttest scores for KE-GD and LV-GD are of different scales. (*p* < .10: boldface; *p* < .05: boldface and starred; NLG: normalized learning gain; CT/EXP: control/experimental condition.)

To address the lack of validity of LV-GD in the control condition in two datasets above, we conducted further investigation on the 19F dataset where LV-GD showed the weakest association with learning. We wondered whether the bottleneck lay in the input detector KE-GD. If the gaming labels (i.e., values of the dependent variable for fitting LV-GD) were too noisy, it would be hard to get accurate tendency estimates by any means. If we decompose a gaming label, it is the union of 13 gaming labels from 13 gaming patterns in KE-GD. Could some of the patterns in some formats be better considered as not gaming in our control condition? In other words, there might be deeper task format effects in students’ interaction patterns. We conducted another exploratory data analysis where we examined the associations between detected gaming proportions of each of the patterns with learning per format. We used a *local* normalized learning gain computed using tasks related to a specific format rather than all tasks in the pretest and posttest.

The results in Table 6 suggest that on different formats, the same gaming pattern could be helpful or harmful for learning; although the statistical significance (and strengths) of the correlations warrants caution in interpretation and further investigation, the current results do hint at deeper format effect for detected gaming. We updated the detected gaming labels from KE-GD in the control condition by using the union of only the patterns that were negatively associated with learning (regardless of statistical significance) for each format while maintaining the labels of the experimental condition. This was a change in the dependent variable rather than the predictors in LV-GD. We used the updated dataset to fit new LV-GD variants, referred to as LV-GD-PR (where PR stands for pattern reduction), and estimated gaming tendencies for the control condition and the overall dataset. Table 7 shows that LV-GD-PR achieved acceptable validity for the control condition and also boosted the validity for the overall dataset compared to LV-GD and KE-GD. We leave for future work to further improve and test this local refinement method.

**Table 6:** Correlations between proportion of each gaming pattern detected by KE-GD and local normalized learning gains in the control condition in the 19F dataset. Pattern #8 was omitted due to its absence. (+ : rho > 0, − : rho < 0, *p* < .10: *boldface and italicized*)

Format	1	2	3	4	5	6	7	9	10	11	12	13
Avg prop	.01	.08	.01	.00	.13	.00	.01	.01	.01	.00	.01	.02
Table	− .17	+ .09	− .05	− .12	+ .03	*** − .22***	− .11	− .05	− .09	+ .06	− .12	+ .01
Menu	+ .04	− .06	** + *.23***	− .18	− .07	− .13	+ .04	− .01	− .06	− .20	*** − .26***	− .19
Flip-M	+ .09	− .15	+ .07	na	− .18	− .01	+ .03	+ .02	− .13	+ .09	− .22	+ .04

**Table 7:** Associations between gaming (from KE-GD, LV-GD, or LV-GD-PR) with learning

Detector	All		CT (control condition)	
	Cor w/ NLG	Post ~ Pre + G	Cor w/ NLG	Post ~ Pre + G
KE-GD	− .02(.86)	0.07(.69)	.14(.25)	0.34(.11)
LV-GD	** − .18(.04)**	*** − 0.04(.09)***	− .02(.86)	− 0.01(.62)
LV-GD-PR	** − .27(.00)**	** − 0.07(.01)**	*** − .23(.06)***	** − 0.06(.04)**

Rho and *p* values are reported for correlation with normalized learning gains; coefficients and *p* values of Gaming variables are reported for regression (*p* < .10: boldface and italicized; *p* < .05: boldface)

## Reliability of LV-GD

In this section, we examined the reliability of LV-GD by seeing whether gaming levels estimated from a set of items correlate or equate well with those estimated from another set of items for the same set of students. This analysis is important for two reasons. First, reliability can further support the validity of LV-GD. Specifically, reliability can support its underlying assumption on the existence of a student-level trait-like component of the gaming construct stable across contexts (as supported by some prior work reviewed in Sect. 1.4), and the ability of LV-GD to extract this component. We expect that gaming estimates from LV-GD demonstrate stability over different subsets of items due to controlling for contextual factors. Second, reliability can also demonstrate the ability of LV-GD to generalize or extrapolate to new contexts. Specifically, we examined whether the estimated gaming tendencies extrapolate to new contexts for the same students.

We used three data splitting methods and two metrics to examine reliability. Different data splitting methods are relevant to different meaningful problem selection designs or intervention designs. Each method corresponds to one way of splitting the data into two sets of non-overlapping items, i.e., two buckets. We only kept overlapping students shared by both buckets for analysis. The three data splitting methods are temporal split, one-vs-rest format split, and random format split. Details will be explained in the following subsections. We measured reliability by two metrics: the correlation between the gaming levels of two buckets (using Spearman correlation) as the primary metric, and the equality between the gaming levels of two buckets (using a paired t-test) as a secondary metric. The correlation metric tells whether a student with a higher gaming level on a set of items still has a higher gaming level on another set of items, i.e., whether the relative gaming levels hold. Such a correlation analysis was used in prior work investigating the reliability of a gaming detector (Muldner et al. 2011). The equality metric tells whether a student’s gaming level on a set of items stays the same on another set of items, i.e., whether the absolute gaming levels hold. This metric may be relevant in some scenarios, such as when system designers need to derive thresholds from absolute gaming levels on a set of items to trigger interventions for other items.

We were primarily interested in whether LV-GD could reach acceptable reliability, i.e., having positive correlations and reaching a significance level of 0.10 for both the correlation and equality analyses. We also compared the reliability between KE-GD and LV-GD and investigated factors affecting the (relative) reliability of LV-GD to obtain a deeper understanding of the two detectors. We examined reliability over the three datasets introduced earlier. In the following subsections, we explained the details of the data splitting and results under each data splitting; then, we investigated the factors that affect the reliability of both detectors.

### Temporal split

Here, we split each student’s temporally ordered data into two buckets by the midpoint of their sequence (i.e., each bucket has the same number of attempts), and then examined the correlation and equality between the gaming levels of two buckets. We expect that gaming levels estimated by LV-GD from earlier interactions correlate with and stay the same as those from later interactions, because LV-GD controls for contextual factors that may vary across time for estimating stable trait-like gaming tendencies. Table 8 shows the result. Overall, LV-GD reached reliability in all datasets, while KE-GD reached reliability in correlation but not always in equality over the datasets. More specifically, LV-GD reached positive significant correlations (*p*s < 0.001) and equality (*p*s > 0.10) over three datasets. Although KE-GD also reached positive significant correlations (*p*s < 0.001) over three datasets, the equality was violated in two datasets (*p*s < 0.10). To our surprise, the rho values of KE-GD were larger than those of LV-GD. We investigate this in Sect. 4.4.

**Table 8:** Reliability under the temporal split for KE-GD and LV-GD across three datasets (correlation *p* < .10: boldface; paired *t*-test *p* > .10 boldface)

Data	Detector	Avg Gaming Level		Correlation			Paired *t*-test	
		Bucke*t* 1	Bucke*t* 2	rho	*p*	Correlated	*p*	Equal
19F (*N* = 129)	KE-GD	0.16	0.20	**.50**	** < .001**	**Yes**	< .001	No
	LV-GD	1.23	1.27	**.35**	** < .001**	**Yes**	**.71**	**Yes**
20S (*N* = 222)	KE-GD	0.16	0.17	**.41**	** < .001**	**Yes**	.09	No
	LV-GD	1.51	1.44	**.32**	** < .001**	**Yes**	**.61**	**Yes**
21F (*N* = 99)	KE-GD	0.17	0.15	**.45**	** < .001**	**Yes**	**.25**	**Yes**
	LV-GD	1.67	1.62	**.34**	** < .001**	**Yes**	**.82**	**Yes**

### One-vs-rest format split

Here, we split each student’s data into two buckets by putting all the data of one format into one bucket, and the remaining three or four formats into another, and then examined the correlation and equality between the gaming levels of two buckets. We repeated this process for each format. The estimated gaming levels by LV-GD from a set of formats should correlate with and stay the same as those from a new format (or estimated gaming levels from a format correlate with and stay the same as those from a set of new formats), because LV-GD controls for contextual factors such as formats for estimating trait-like gaming tendencies independent of formats. This splitting poses a higher challenge to extrapolate than the temporal split where both buckets may still share some formats.

Table 9 shows the result. Overall, LV-GD reached reliability in the majority of cases and in more cases than KE-GD over the datasets. Looking into LV-GD first, the correlations were all positive and most of the time (11/13 = 85%) the significance levels were met (*p*s < 0.10), except for two cases where sample sizes were small. Equality was met (*p*s > 0.10) the majority of the time (7/13 = 54%), except for some cases, especially where the new format was table or text. In this data splitting setting, the reliability of LV-GD in terms of correlation appears to require having sufficient data to estimate the normative behaviors of the population (i.e., the fixed effects) in a bucket in order to extrapolate to another bucket. Also, the reliability of LV-GD in terms of equality is challenged when the new format is of certain formats (e.g., table). Looking into KE-GD in comparison with LV-GD, the correlations were all positive and the significance levels were met (*p*s < 0.10) in one fewer case than LV-GD (10/13 = 77%), where the exceptions did not necessarily have small sample sizes; equality was never met (*p*s < 0.10).

**Table 9:** Reliability under the one-vs-rest format split for KE-GD and LV-GD across formats on three datasets (correlation *p* < .10: *boldface and italicized*, *p* < .05: boldface; paired *t*-test *p* > .10 boldface)

Data	Bucket 1 versus Bucket 2	*N*	Correlation				Paired *t*-test	
			KE-GD		LV-GD		KE-GD	LV-GD
			rho	*p*	rho	*p*	*p*	*p*
19F	Text versus Rest	59	**.27**	**.04**	.20	.13	< .001	.06
	Table versus Rest	118	**.20**	**.03**	**.20**	**.03**	< .001	< .001
	FlipMenu versus Rest	109	**.31**	** < .001**	**.23**	**.02**	< .001	**.50**
	Menu versus Rest	117	**.42**	** < .001**	**.29**	**.001**	< .001	**.64**
	Avg over above four cases	–	.30	**–**	.23	**–**	–	**–**
20S	Text versus Rest	100	.12	.23	**.24**	**.02**	.006	**.35**
	Table versus Rest	182	.08	.29	**.15**	**.04**	< .001	.006
	Flip-menu versus Rest	112	**.52**	** < .001**	**.26**	**.005**	< .001	.01
	Menu versus Rest	110	**.47**	** < .001**	**.31**	**.001**	< .001	**.70**
	Avg over above four cases	–	.30	**–**	.24	**–**	–	**–**
21F	Text versus Rest	53	**.32**	**.02**	**.49**	** < .001**	.053	.053
	Table versus Rest	78	**.25**	**.03**	***.22***	***.051***	< .001	.03
	Flip-menu versus Rest	55	**.27**	**.046**	**.39**	**.004**	< .001	**.78**
	Menu versus Rest	27	.11	.58	.23	.25	< .001	**.75**
	Animation versus Rest	53	**.51**	** < .001**	**.42**	**.002**	.059	**.65**
	Avg over above five cases	–	.29	**–**	.35	**–**	–	**–**
All	% cases correlated or equal	–	10/13 = 77%		11/13 = 85%		0%	7/13 = 54%

### Random format split

Lastly, we split each student’s data into two buckets by randomly selecting half of the formats and putting them into one bucket, and putting the remaining formats into another bucket, and then examined the correlation and equality between the gaming levels of two buckets. Note that for different students, different formats might have been selected for a bucket (e.g., student A may have table and text formats in the first bucket while student B may have menu and text formats in the first bucket). This data splitting allows for seeing how well a detector extrapolates from a set of formats to another set of formats, i.e., whether a student who has a higher estimated gaming level on some formats also has a higher estimated gaming level on other formats, and whether a student’s estimated gaming level on some formats stays the same on other formats. This is relevant when different students are given formats in different orders in a tutor. This setting is different from the one-vs-rest format split where students in the same bucket have the same format coverage which is relevant when students are given formats in the same order in a tutor. Also, this way of splitting (i.e., format-stratified) places a higher challenge to test reliability (or extrapolate) than randomly splitting the data by problems (i.e., problem-stratified) used in prior work (Muldner et al. 2011). In problem-stratified splitting, both buckets likely share formats for the same student and thus a detector can utilize data on a format from one bucket to extrapolate to the same format in another bucket for the student, which is not the case for format-stratified splitting.

Table 10 shows the result. Overall, LV-GD reached reliability in all datasets, while KE-GD did not reach correlation-based reliability in any dataset and reached equality-based reliability in two datasets. More specifically, LV-GD reached positive significant correlations (*p*s < 0.01) and equality (*p*s > 0.10) in all datasets. However, KE-GD did not reach positive significant correlation in any dataset and even had a negative significant correlation in the 19F dataset (*rho* =  − 0.20, *p* = 0.03); the equality was met in two datasets (*p*s > 0.10) but was violated in the 19F dataset (*p* = 0.04).

**Table 10:** Reliability under the random format split for KE-GD and LV-GD over three datasets (correlation *p* < .10: boldface; paired *t*-test *p* > .10 boldface)

Data	Detector	Avg Gaming Level		Correlation			Paired *t*-test	
		Bucke*t* 1	Bucke*t* 2	rho	*p*	Correlated	*p*	Equal
19F (*N* = 118)	KE-GD	0.19	0.15	− .20	.03	No	.04	No
	LV-GD	1.23	1.28	**.24**	**.009**	**Yes**	**.57**	**Yes**
20S (*N* = 182)	KE-GD	0.16	0.15	.06	.41	No	**.75**	**Yes**
	LV-GD	1.36	1.59	**.25**	** < .001**	**Yes**	**.15**	**Yes**
21F (*N* = 79)	KE-GD	0.17	0.17	.11	.34	Yes	**.84**	**Yes**
	LV-GD	1.33	1.63	**.32**	**.005**	**Yes**	**.19**	**Yes**

### The effect of format context similarity

Across the above three data splitting methods, LV-GD reached reliability in all cases (under temporal split and random format split) or in the majority of cases (under one-vs-rest format split), and reached reliability in more cases than KE-GD. Yet we also noticed the relative performance of LV-GD in comparison with KE-GD varied across the splitting methods, and KE-GD sometimes reached higher correlation rho values than LV-GD (e.g., all cases under temporal split and some cases under one-vs-rest format split). Thus, we conducted further investigation into factors that affect the (relative) reliability of LV-GD. We hypothesized that *format context similarity* within a bucket across students and between buckets for a student may affect the (relative) reliability of LV-GD. We broke down format context similarity into two kinds: between-student format context similarity and within-student format context similarity. More specifically, in this subsection, a *context* is defined with respect to the data of a student in a bucket (and refers to a bucket in a particular split such as *Text vs. Rest* in the one-vs-rest format split), and is represented as a *context vector* whose dimensions are the proportions of attempts over different formats of the student in the bucket. For example, if the data of a student in a bucket consists of 40% of the text format, 10% of the table format, 30% of the flip-menu format and 20% of the menu format, then the context vector is (0.4, 0.1, 0.3, 0.2). *Between-student format context similarity* indicates the similarity between the context vectors of different students in the same bucket; it is defined for a student pair within a bucket at the lowest level using the cosine similarity between the context vectors, and is then aggregated for a bucket, a dataset, or multiple datasets by computing the average over student pairs, buckets, or datasets, respectively. *Within-student format context similarity* indicates the similarity between the context vectors of different buckets of the same student; it is defined for a student across two buckets at the lowest level using the cosine similarity between the context vectors, and is then aggregated for a dataset or multiple datasets by computing the average over students or datasets, respectively. These two kinds of similarity measures can be further averaged to obtain a single format context similarity measure.

Table 11 presents the detailed statistics of format context similarity for each dataset for each data splitting method, and Table 12 presents the aggregated statistics connecting both reliability and format context similarity measures over the three datasets. We focus on Table 12 for obtaining insights. In general, the advantage of LV-GD over KE-GD (especially in correlation) increases from temporal split to random format split, as the format context similarity decreases. Looking closer into where LV-GD had the greatest advantage over KE-GD, the random format split, the between-student format context similarity here is the lowest and is lower than 0.50, meaning that the contexts between students within a bucket are actually dissimilar. In this case, KE-GD could not obtain reliable relative estimates of gaming levels at all (0% of cases correlated) and only obtained moderately reliable absolute estimates (67% of cases equal), while LV-GD could still reliably estimate students’ relative and absolute gaming levels (100% of cases correlated and 100% of cases equal). Looking closer into where LV-GD had the worst performance, the one-vs-rest format split, this is the only setting where two buckets do not share formats in any way either from the same student or from other students. In this setting, it is hard for both detectors to extrapolate to completely new formats, but LV-GD could still estimate the relative and absolute gaming levels well in the majority of cases and in more cases than KE-GD, and KE-GD performed extremely badly in the equality metric—the absolute gaming level estimates from one bucket could not be extrapolated to the other bucket in any case.

**Table 11:** Format context similarity under different data splitting methods on three datasets

Data splitting method	Data	Betw-student format context similarity			Within-student format context similarity M(SD)	Avg of similarity measures
		Bucket 1 M(SD)	Bucket 2 M(SD)	Avg of buckets		
Temporal split	19F	.68(.30)	.68(.32)	.68	.76(.26)	.72
	20S	.53(.35)	.40(.41)	.47	.67(.32)	.57
	21F	.53(.34)	.39(.38)	.46	.70(.31)	.58
One-vs-rest format split	19F	1(0)	.86(.15)	.93	0(0)	.47
	20S	1(0)	.69(.31)	.85	0(0)	.43
	21F	1(0)	.68(.28)	.84	0(0)	.42
Random format split	19F	.48(.39)	.35(.40)	.42	0(0)	.21
	20S	.36(.39)	.30(.43)	.33	0(0)	.17
	21F	.34(.36)	.26(.38)	.30	0(0)	.15

A similarity value is measured by the cosine similarity between two context vectors and ranges from zero to one with higher value indicating higher similarity

**Table 12:** Reliability and format context similarity by the averages over the datasets

Splitting method	Cor. (avg rho)		% cases correlated		% cases equal		Format context similarity		
	KE-GD	LV-GD	KE-GD (%)	LV-GD (%)	KE-GD (%)	LV-GD (%)	Betw-stu	With-stu	Avg
Temporal	.45	.34	100	100	33	100	.54	.71	.62
One-vs-rest	.30	.27	77	85	0	54	.87	0	.44
Random	− .01	.27	0	100	67	100	.35	0	.18

Altogether, format context similarity indeed affects the relative reliability of LV-GD compared to KE-GD. KE-GD relies more heavily on format context similarity to exhibit reliability or extrapolate; its gaming level estimates likely reflect the characteristics of the contexts. On the other hand, LV-GD is much less dependent on format context similarity to exhibit reliability or extrapolate; its gaming level estimates reflect students’ intrinsic trait-like gaming tendencies. When format context similarity is high, KE-GD can reach reliability comparable with (or even better than) LV-GD in terms of the strength of correlations, but LV-GD reaches reliability in more cases than KE-GD for both correlation and equality, and is more robust against challenges posed by context dissimilarity than KE-GD. Meanwhile, we also noticed that the reliability of both KE-GD and LV-GD has a decreasing trend with the decrease of format context similarity, suggesting some degree of influence from the contexts on the gaming estimates.

Looking across analyses on reliability in this section, results consistently demonstrate the reliability of LV-GD across different data splitting methods and datasets. These results further support the validity of LV-GD. More specifically, results support the trait-like property of the gaming construct besides the state-like property, and the ability of LV-GD to extract this trait-like component. In addition, results also show the ability of LV-GD to extrapolate to new situations (e.g., new problems with seen or unseen formats) in terms of its gaming tendency estimates.

## Applications of LV-GD

In this section, we demonstrated three applications of the estimated gaming tendencies from LV-GD: to study intervention effects on gaming (i.e., whether there was a difference in the gaming levels between the two conditions from our experimentation with the tutor), to explore the relation between gaming and motivation, and to understand productive detected gaming behaviors.

### Studying intervention effects on gaming

Our prior work (Huang et al. 2021) showed that intense deliberate practice (experimental condition) led to greater learning outcomes compared to normal deliberate practice (control condition) in the first experiment (19F dataset); we are interested to see whether the intervention also led to higher behavioral engagement, particularly lower levels of gaming the system. We conducted a regression analysis predicting levels of gaming over students given the condition indicator on the three datasets. The two detectors had contradicting results on the 19F and 20S datasets. On the 19F dataset, KE-GD showed that the intervention led to significantly higher levels of gaming while LV-GD showed that there was no statistical difference (Table 13 the 2nd column). The suggested intervention effect of increased gaming levels by KE-GD contradicted the previously validated intervention effect of improved learning, since higher levels of gaming are usually associated with lower learning. Thus, LV-GD more accurately revealed the intervention effect on this dataset. We hypothesized that this could be due to KE-GD not being able to account for the task format effect. We computed the proportion of highly gamed formats over actions and the normalized learning gain per student per condition. We found that the EXP condition had a higher average proportion of highly gamed formats (Table 14 the 2nd column), consistent with our hypothesis. On the 20S dataset, KE-GD showed that the intervention led to significantly lower levels of gaming while LV-GD showed that there was no statistical difference (Table 13 the 3rd column). However, both conditions have similar normalized learning gains, and the control condition had a much higher average proportion of highly gamed formats (Table 14 the 20S columns). This again suggests that KE-GD provided biased gaming assessment by using direct proportion of gaming without accounting for formats. This set of analyses shows that LV-GD more accurately revealed intervention effects on gaming than KE-GD in our experiments.

**Table 13:** Intervention effects on gaming examined by regression predicting gaming proportions or tendencies given the condition variable (Control: 0, Experimental: 1; *p *< .05: boldface)

Detector	19F	20S	21F
KE-GD	***b* = 0.02, *p* = .03**	***b* = − 0.09, *p* < .001**	*b* = 0.03, *p* = .15
LV-GD	*b* = − 0.05, *p* = .68	*b* = 0.03, *p* = .79	*b* = 0.16, *p* = .48

Coefficients of the condition variable are reported

**Table 14:** The proportion of highly gamed formats (PHGF) in actions and normalized learning gain per condition

Cond	19F		20S		21F	
	PHGF	NLG	PHGF	NLG	PHGF	NLG
CT	.38(.22)	.16(.29)	**.31(.22)**	.12(.34)	.16(.17)	.14(.20)
EXP	**.44(.09)**	**.24(.28)**	.06(.13)	**.14(.39)**	**.40(.16)**	**.15(.25)**

Mean and SD are reported. Higher values are in boldface

### Studying the relation between motivation and gaming

The investigation of the relation between motivation and gaming contributes to understanding why students game and developing behavioral measures of motivation. Prior work (Baker et al. 2008a, b) indicated that students’ attitudes and interest toward the domain was related to detected (observed) gaming frequency. More recent work (Dang and Koedinger 2019) identified strong associations between several motivational measures and estimated gaming tendencies. Our investigation of the relation between motivation and gaming adds to the limited empirical evidence in this space. On our datasets, motivational surveys with four scales (Table 15) were collected at the first and the last sessions of each month-long experiment. Responses for each scale were averaged to present students’ motivation along the scale for a pre-survey or a post-survey. Table 16 shows correlations between motivational measures from surveys measured at the beginning of the first session and estimated gaming tendencies over students. Only perceived competence in math showed consistent significant correlations with gaming and only in the experimental condition across three datasets; the sign of the correlations was negative as theoretically predicted. These correlations did not appear to be due to students’ abilities approximated by pretest scores, because we did not find correlations between pretest scores and gaming tendencies. To understand why perceived confidence was only associated with gaming in the experimental but not in the control condition, we compared objective difficulties measured by the proportion correct of first attempts and subjective difficulties measured by the difference between the final and the initial values of perceived confidence between the conditions (Table 17). We found that the experimental condition tended to have lower objective difficulties but higher subjective difficulties. We discuss the results in Sect. 6.3.

**Table 15:** Motivational survey inventory (7-point Likert rating)

Scale	Question
Perceived competence in math	How good at math are you?
	Compared to most of your other school subjects, how good are you at math?
Math utility value	How important is it to you to learn math?
	How important do you think math will be to you in the future?
Interest in math	How interesting is math to you?
Interest in tutor	How excited are you to do math on a computer?

**Table 16:** Correlations (Spearman’s *rho*) between motivational measures from surveys and estimated gaming tendencies (*p* < .10: boldface)

Scale	Cond	19F	20S	21F
Perceived competence in math	CT	− .00(.97)	− .10(.31)	− .03(.84)
	EXP	** − .26(.046)**	** − .18(.05)**	** − .32(.02)**
	All	− .11(.20)	** − .15(.03)**	− .12(.25)
Math utility value	CT	− .11(.38)	.00(.98)	** − .31(.04)**
	EXP	.09(.50)	− .03(.78)	− .17(.22)
	All	.01(.94)	− .02(.78)	** − .21(.04)**
Interest in math	CT	.04(.73)	− .03(.75)	− .05(.73)
	EXP	− .07(.60)	− .13(.18)	− .11(.41)
	All	.02(.87)	− .08(.21)	− .04(.66)
Interest in tutor	CT	.11(.37)	− .03(.75)	− .01(.97)
	EXP	.06(.64)	− .06(.55)	− .01(.93)
	All	.12(.19)	− .04(.54)	− .01(.93)
Pretest	CT	− .04(.74)	− .01(.91)	− .06(.68)
	EXP	− .01(.92)	− .03(.74)	− .06(.66)
	All	− .01(.90)	− .04(.61)	− .07(.52)

Correlations with pretest scores were added for contrast

**Table 17:** Proportion correct of first attempts (objective difficulties) and the difference of perceived competence in math between the final value and the initial value (subjective difficulties; $\Delta$ PC)

Cond	19F		20S		21F	
	prop cor	$\Delta$ PC	prop cor	$\Delta$ PC	prop cor	$\Delta$ PC
CT	.60(.17)	**.02(.86)**	.60(.14)	** − .03(.83)**	.61(.16)	**.02(1.11)**
EXP	**.62(.10)**	− .22(.91)	**.69(.11)**	− .09(.86)	**.70(.10)**	− .22(.98)

Mean and SD are reported. Higher values are in boldface

### Understanding productive detected gaming

In this section, we demonstrate a preliminary case study where we used LV-GD to help select cases for understanding productive detected gaming behaviors and generate insights into improving action-level gaming detection. In Sect. 2 on 19F dataset, we observed a significant crossover interaction where the control condition exhibited a positive association between gaming levels by KE-GD and learning (Fig. 5), while both conditions showed negative associations using LV-GD (Fig. 10) consistent with the theoretical construct of gaming. The difference in the judgements between KE-GD and LV-GD—more specifically, when KE-GD asserts high gaming levels but LV-GD asserts low gaming levels—likely indicates productive detected gaming, because low gaming levels asserted by LV-GD were associated with high learning gains. We focused on the 19F dataset in this analysis. Details are as follows.

**Fig. 11:**
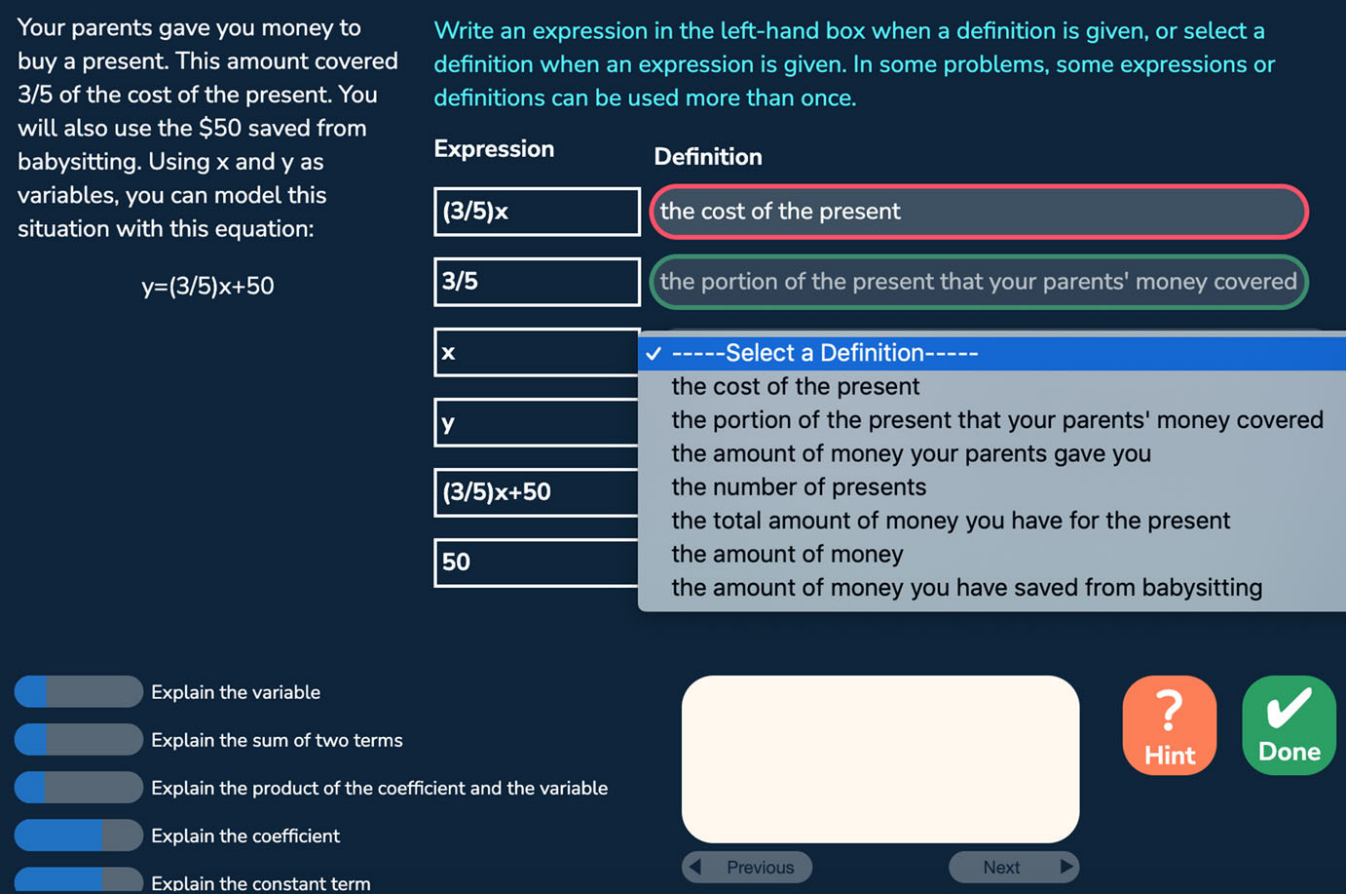
The chosen menu problem with a high number of attempts detected as gaming from a student with a high level of productive detected gaming

First, we identified a representative case of productive detected gaming. We used medians to separate students into *low* and *high* groups for detected gaming by KE-GD, gaming tendencies by LV-GD, and normalized learning gains (NLGs). We considered students with high detected gaming and high NLGs, resulting in 31 students. We then utilized LV-GD to keep only those with low gaming tendencies, which reduced the sample size by 77% resulting in seven students. Then we considered students from the control condition and selected the one with the highest NLG. Next, we selected a problem for the chosen student. We focused on menu problems for their highest detected gaming levels and picked one with the highest number of attempts detected as gaming for the student (Fig. 11). This problem was the eighth menu problem she saw.

Second, we conducted observations of the selected case. Table 18 shows her first seven actions. She first chose the easiest steps *3/5* and *50* (57% and 67% correct for all students in such steps) and got them correct. She then accessed the remaining first step in the interface, *(3/5)x*, spent 17 s and then selected an option which was wrong but still relevant. After 8 s, she chose another option which was wrong but not completely off since the description appears in the text right after 3/5, part of the expression *(**3**/5)x*. Having failed twice on this hardest step (34% correct), she switched to the second step in the interface, *x*. After 11 s, she chose an option which was wrong but on the right track because it refers to (3/5)*x* that involves *x*. After 12 s, she selected an option which was farther away from the correct option but not as irrelevant as some other options. Having failed twice on this step, which is the second hardest (42% correct), she paused for 9 s and jumped to the fourth step in the interface (3/5)*x* + *50*, used the option just selected as the answer, and got it right.

**Table 18:** The selected case representing productive detected gaming behaviors

ID	DG	Step	Position	Result	Second	Answer	Correct ans
1	0	3/5	2rd step	Correct	15	The portion of the present that your parents’ money covered	
2	0	50	6th step	Correct	18	The amount of money you have saved from babysitting	
3	1	(3/5)x	1st step	Wrong	17	The total amount of money you have for the present	The amount of money your parents gave you
4	1	(3/5)x	1st step	Wrong	8	The cost of the present	
5	1	x	3rd step	Wrong	11	The amount of money your parents gave you	The cost of the present
6	1	x	3rd step	Wrong	12	The total amount of money you have for the present	
7	1	(3/5)x + 50	5th step	Correct	9	The total amount of money you have for the present	

Third, we generated hypotheses about productive detected gaming and recommendations to refine KE-GD based on its detected gaming patterns. Table 19 shows the detected gaming patterns by KE-GD in the seven actions. In contrast, Table 20 shows our hypothesized productive meta-cognitive strategies for detected gaming actions (actions 3–7) and actions around them (actions 1–2). Specifically, actions 3–7 may be considered as a sample of productive detected gaming behaviors, which all involve durations that suggest deliberation. We generated two recommendations to refine KE-GD. First, at a step level, more contextual information of current actions may be considered in defining gaming patterns, such as time spent on an action or the difficulty of a step. Second, looking at behaviors at a problem level beyond current actions may be helpful. For example, a likely productive meta-cognitive strategy in earlier or later actions within the problem (e.g., select an easier step to work on first) may increase the likelihood of the student being productively engaged in current actions.

**Table 19:** The gaming patterns detected by KE-GD on actions in Table 18

Action IDs	Pattern ID	Matched gaming pattern in KE-GD
3 → 4 → 5	9	**incorrect** → [similar answer] & **incorrect** → [switched context before correct] & **incorrect**
4 → 5 → 6	10	**incorrect** → [switched context before correct] & **incorrect** → [similar answer] & **incorrect**
5 → 6 → 7	3	**incorrect** → [similar answer] & **incorrect** → [same answer in different context] & **attempt** (correct or incorrect)

[similar answer] means an answer was similar to the previous action (Levenshtein distance of 1 or 2). We have adapted the computation of Levenshtein distance for texts by counting overlapping words between two phrases after removing stop words. If the number of overlapping words is no less than half of the number of words of the shorter phrase, then the two phrases are considered as being similar

Actions of giving answers (incorrect or attempt) are in boldface

**Table 20:** Hypothesized productive meta-cognitive strategies on actions in Table 18. All except the first two actions were detected as gaming by KE-GD in Table 19

Action IDs	Hypothesized productive meta-cognitive strategies
1,2	Select an easier step to work on first, with duration suggesting deliberation
3,4,5,6	Gives answers relevant to the correct answer, with duration suggesting deliberation
4	Locate relevant information in the problem statement, with duration suggesting deliberation
3 → 4 → 5, 5 → 6 → 7	Move on to another step after multiple failures (on a hard step), with duration suggesting deliberation
6 → 7	Move on to the step that matches a previously wrong answer (on a hard step), with duration suggesting deliberation

## Conclusions and discussion

In this paper, we present an approach using latent variable models for more valid and robust gaming assessment and demonstrate its validity, reliability, generalizability as well as practical usability through a comprehensive evaluation on multiple datasets. We started with applying a previously validated, winning gaming detector, KE-GD, to a dataset collected from an algebra tutor with varying task designs and sequencing across conditions. However, the detected gaming was not associated with learning overall and was even associated with better learning in the control condition, challenging its validity in our context. We identified contextual factors that capture the normative behaviors of the population that might induce bias and might explain this lack of association. We then integrated the contextual factors through LV-GD, a statistical model, where detected gaming from KE-GD is predicted by both contextual factors and students’ intrinsic gaming tendencies. LV-GD generates gaming measures as student-level latent gaming tendencies, each of which captures the degree of deviation of a student’s behavior from normative behaviors of the population in the same contexts. We evaluated LV-GD on three datasets collected from different populations and versions of the system. LV-GD consistently outperformed KE-GD in validity in terms of association between gaming and learning, suggesting that it effectively isolates unproductive detected gaming behaviors. LV-GD also showed higher reliability (measured by consistencies of gaming estimates from different data subsets). Across the analyses, LV-GD demonstrated generalizability in various ways: its structure generalized to new datasets (Sect. 3) and its latent tendency estimates generalized to new contexts (including to new formats; Sect. 4). LV-GD also afforded high practical utility: it more accurately revealed intervention effects on gaming, revealed a correlation between gaming and perceived competence in math, and helped understand productivity detected gaming behaviors.

Our work makes three key contributions to the field of behavior modeling. First, we introduce a general cost-effective approach using latent variable models (LVMs), to adapt an existing detector to new contexts by integrating contextual factors not originally considered. Most related work has focused on showing that a previously developed detector works in a new context, and in terms of accurately predicting human labels; it is rare to encounter a work that reports that a previously validated detector does not work in a new context in terms of identifying behaviors unproductive for learning, and demonstrates how to adapt it to the new context. Although we only demonstrated applying our approach to one detector, theoretically our approach can be applied to any behavior detector due to the statistical modeling framework. In addition, our approach does not require additional human labeling or complex feature engineering, which may be attractive for the learning engineering community to minimize adaptation efforts. For example, the knowledge level effect on detected gaming is incorporated through practice opportunity counts without an additional process to estimate dynamic knowledge as in Baker et al. (2008a, b) and Walonoski and Heffernan (2006a). The format as a unit of context can be easily operationalized in other datasets and can be replaced by other reasonable units available in a dataset, such as lessons, topics or problem types, thanks to the flexibility of our approach. Second, we show the value of latent trait-level assessment—different from the dominant observed action-level assessment—and open doors for model transfer from the knowledge modeling field where LVM flourishes (Desmarais and Baker 2012; González-Brenes et al. 2014). Specifically, we showed that a latent trait-level assessment through looking across contexts and across students provided a more valid and robust gaming assessment against context variations than an observed action-level assessment from a previously winning detector. Our examination, especially the reliability of LV-GD, also added evidence for the trait-like property (besides the state-like property) of the gaming construct. Third, we showed the importance of establishing validity in a new context when applying a behavior detector to the context. We should not assume that validity is met in a new context and should adapt the detector when validity is not met. Overall, the implication of our work is not that LV-GD should replace existing detectors, but rather, LV-GD or more generally the LVM approach can be used to *enhance* existing detectors, especially when they lack validity in a new context. Our work may help advance the field by increasing cross-system transfer as well as building more valid and robust behavior detectors. Below, we discuss more in depth our work in relation to existing research, while also pointing out limitations and future work.

### Validity and generalizability of LV-GD

The validity of LV-GD, especially in comparison with KE-GD, is supported by various aspects in this work, including the association between gaming and learning, reliability, and generalizability. We did not examine its predictiveness on human labels typically used as the ground truth of gaming behaviors and primary evaluation of a gaming detector. This is a feature rather than a limitation of LV-GD: it builds on human labels indirectly and can reduce bias in the labels. It builds on human labels indirectly because LV-GD predicts the gaming labels of KE-GD, which has been shown to predict human labels well. It can reduce bias in the labels by integrating contextual factors not considered in the original human labeling processes but may help differentiate unproductive detected gaming from productive detected gaming. As validity is typically established incrementally through an accumulation of supporting evidence, there is a need and also room to further improve the validity of LV-GD, as discussed below.

The results of the relation between motivation and gaming appeared to challenge the validity of LV-GD at first glance. We found a negative correlation between perceived competence in math and gaming in the experimental condition, consistent with the reported negative correlation between self-efficacy in math and gaming in the work of Dang and Koedinger (2019). We did not, however, find any correlations between other motivational measures and gaming, such as students’ interest toward the domain and gaming (Baker et al. 2008a, b; Dang and Koedinger 2019), or any correlations in the control condition.^4^ Rather than prematurely attributing the general lack of correlation between motivation and gaming to the lack of validity of LV-GD, we hypothesize several reasons. First, there may be covariates, interactions between different student attributes (measured or unmeasured in the current study) or between student attributes and system attributes not considered in simple zero-order correlations we did here. For example, Dang and Koedinger (2019) had controlled for gender, ethnicity, and free/reduced lunch status in all their reported partial correlations. They also found that gaming estimates using only non-highly gamed materials were significantly related to all targeted motivation measures, which was not the case of highly gamed materials. Second, our motivational survey was not designed to study motivational factors underlying gaming and only measured a small set of constructs; it is possible that the gaming estimates of LV-GD correlate with some unmeasured motivational constructs. Lastly, there is still a lack of empirical evidence and theory of the relation between motivation and gaming. Altogether, we think this set of results do not suffice to challenge the validity of LV-GD; further investigation is needed to understand motivational factors that underlie gaming.

We did not find an association between pretest scores and gaming tendencies from LV-GD (Table 15), whereas prior studies have shown that lower prior knowledge levels were associated with higher gaming frequencies (Baker et al. 2004a, b; Mogessie et al. 2020). We think that the association with lower prior knowledge is not implied in the standard definitions of gaming (Baker et al. 2006a, 2008a, b), and some work found that gaming could occur on skills where students had high estimated knowledge (Baker, Corbett and Koedinger, 2004). LV-GD includes pretest scores and relevant interactions as predictors for detected gaming and thus can be viewed as being designed to extract latent gaming tendencies that are not (primarily) triggered by prior knowledge, but by other factors such as students’ motivation or metacognition. Such gaming measures may be useful for system designs or analyses that are concerned with motivation or metacognition. However, our approach is flexible in that one could drop the pretest score-related predictors if they are interested in gaming (primarily) triggered by prior knowledge.

Our reliability analysis supports the trait-like property of the gaming construct (besides the state-like property), yet it is still unclear the causal factors for this trait-like property. Possible causal factors to be tested include students’ certain motivational attributes, meta-cognitive attributes, as well as domain-independent skills or learning abilities. Identifying such factors may help design interventions to reduce gaming.

In some contexts (e.g., the control condition), the standard full formulation of LV-GD (Table 2 ID = 5) did not reach statistical significance for the association between gaming and learning, although its associations were still better than those of KE-GD. In one of such contexts, we investigated a variant of LV-GD where we integrated deeper format effects, i.e., the interaction between formats and specific interaction patterns, into the labels of the input detector, KE-GD. Our refinement led to acceptable statistical significance and demonstrates the flexibility of our latent variable modeling approach. A next step is to test the robustness of this refinement method and investigate better ways to integrate deeper format effects, such as considering the strength of the correlation (Table 6) when updating the detected gaming labels from KE-GD.

Generalizability is also an important desirable property of behavior detectors. In the current work, we have demonstrated the generalizability of LV-GD in various ways, but there are still a few to be examined and also there is room to improve. First, we have not examined whether a fitted LV-GD from past data can estimate gaming tendencies well for new students. This aspect is especially relevant to online intervention where the system has to react to unproductive detected gaming for new students. In theory LV-GD allows such an extrapolation: before observing any data points of the student, we can assume the average gaming tendency (which is zero) for the student; after observing at least one data point of the student, we can (repeatedly) reestimate the parameters with the accumulated data fitting a new random intercept (i.e., a gaming tendency) for the new student. Second, we have only investigated extrapolating estimated gaming tendencies to an unseen format without using any data points from the unseen format (Sect. 4.2). However, in an online setting, we can utilize the accumulated data of the unseen format: after observing at least one data point of the new format, we can (repeatedly) reestimate the parameters (e.g., gaming tendencies) with a parameter added and fitted for the new format.^5^ A promising modification of LV-GD that enables greater generalizability to new formats is to replace the dummy coded format variables with a variable that describes key properties of formats, e.g., *whether a response set is given or can be easily inferred*. Third, our three datasets are from the same system albeit with changes in the designs, and we need to test the generalizability of LV-GD to other systems. Lastly, we may also test whether our approach can also enhance other behavior detectors.

### Implications for developing gaming detectors

The findings in this work have implications for developing gaming detectors. First, we have identified contextual factors that could be broadly interpreted and considered in the development of gaming detectors. The format factor we identified may imply a general contextual factor—cognitive cost (Flake et al. 2015). Two formats exhibited high detected gaming levels, menu and flipped-menu formats, cohering with prior work that included related task features in the final detectors hinting at the high propensity of the menu or multiple-choice formats for triggering detected gaming (Baker et al. 2008a, b; Walonoski and Heffernan 2006a); this finding also coheres with prior work that reported a high average gaming proportion (13.8%) on a tutor that consists of only multiple-choice format problems (Peters et al. 2018). Such formats appear to require low cognitive cost in making an attempt because a response set is given or can be easily inferred in the task design (e.g., in flipped-menu problems, students can write expressions extracted from a given equation with limited possibilities rather than writing expressions from scratch). When the cognitive cost in making an attempt is low, students may use game-like learning strategies such as a trial-and-error strategy with genuine engagement. Moreover, the interaction between format and pretest scores where menu and flipped-menu formats exhibited significant negative correlations (Fig. 8) suggests that low cognitive cost may also trigger game-like learning strategies for lower-level students to a greater extent than for higher-level students. Thus, it is worth paying more attention to cognitive cost in developing gaming detectors especially for excluding productive detected gaming behaviors. For example, in human labeling processes, we can display information related to the cognitive cost in a current format or problem type, which may include the median time of an attempt and average number of attempts per step of the population, of the low-level students and of the high-level students; if and only if a student deviates much (e.g., in terms of SD) from the (sub) population, we consider the behaviors as gaming.

The format by practice opportunity interaction where the table format exhibited a significant negative correlation (Fig. 9) suggests another general contextual factor—the clarity of the instruction. Examining table problems, we did not find instructions on how to fill in the various cells of the table (Fig. 2) and there are places that may cause confusion. For example, under the column labeled as “Show your work,” it is not clear whether a student could enter *15* + *10* (graded as wrong) instead of 3*5 + *10* (the correct answer). This coheres with prior work suggesting that students gamed more when the presentation is unclear (Baker et al. 2009), and that students may game as a way to obtain worked examples (Shih et al. 2008). When the instruction is unclear, students may use game-like behaviors at the beginning to get to know the format; this kind of difficulty decreases quickly resulting in a fast decrease in detected gaming levels as students become more familiar with the format. Thus, it is worth paying more attention to the clarity of instructions or presentation in developing gaming detectors, especially for excluding productive detected gaming behaviors. For example, in human labeling processes, we can display the interface of the current problem, population statistics (e.g., number of attempts and help requests) at the same or similar opportunities, as well as the student’s deviation from the population, to help make judgments of gaming. A final remark regarding task formats is that in the tutor we studied, the interpretation of task formats requires caution since a task format is not only coupled with a specific interface design (as the name *format* suggests), but also a specific scaffolding design (e.g., fixed or dynamic scaffolding) as well as specific KCs. A future direction is to study them separately through experimentation.

Second, our preliminary case study empowered by LV-GD also points out directions to improve gaming detectors in general (Sect. 5.3). Specifically, we generated two recommendations: considering more contextual information of current actions at a step level and looking at behaviors beyond current actions at a problem level. As a next step, we may inspect more cases and combine qualitative and quantitative analyses to identify common, consistent patterns or characteristics of productive detected gaming behaviors for more robust gaming detection, which is still under-investigated in the field.

Lastly, LV-GD also has the potential to conduct action-level gaming detection. For example, we can first fit an LV-GD model to past data using the full formulation. Then, on a new dataset or a running system, we can apply the fitted model without using the random student intercepts (i.e., student gaming tendencies) to predict how likely an average or typical student will game (as defined by KE-GD) at a current step according to current contextual factors. If a typical student is unlikely to exhibit detected gaming behaviors in a context according to this population-level prediction (with an interval of uncertainty), but a student was detected as gaming (by KE-GD), we will label such behaviors as gaming or activate a pre-designed intervention; otherwise, if the student was detected as gaming but did not deviate much from the norm, we will not label such behaviors as gaming or activate the intervention.

### Implications for system design

Our work also provides implications for system design different from prior work. Past research (Baker et al. 2009) investigating task features that encourage and discourage gaming has generated design recommendations to reduce gaming (e.g., replacing textual references to abstract principles in hints with other ways of communicating abstract principles), but they have not examined whether detected gaming was associated with less learning. In this work, although we also identified task features that triggered high levels of detected gaming, our results suggest that some detected gaming behaviors could actually be productive. Thus, instead of immediately recommending redesigns to reduce gaming, our work encourages further investigation of whether certain task features trigger high levels of *unproductive* detected gaming before redesign decisions. Before the answer is clear, one may consider focusing on improving gaming detection so that it isolates unproductive detected gaming and designing reactive interventions.

Our investigation of the correlation between motivation and gaming (Sect. 5.2) also provides implications for motivational design in the context of intense deliberate practice. We found a negative correlation between perceived competence in math and gaming in the intense deliberate practice condition (the experimental condition) but not in the normal deliberate practice condition (the control condition). We conducted a preliminary exploration and found that the objective difficulty (measured by the proportion correct of first attempts) of the intense deliberate practice condition appeared to be lower than the normal deliberate practice condition but the subjective difficulty (measured by perceived competence in math) of it appeared to be higher. One hypothesis is that the patterns of successes or failures may matter more than the proportion of success for students’ perceived competence. The intense deliberate practice driven by a more fine-grained and larger KC model may have more constantly pushed students to work on their weak spots in new tasks (i.e., put them on the edge of competence), challenging their perceived competence. It may be worth considering letting students occasionally work on already mastered skills to boost their perceived competence, or preparing students better for desirable difficulties or failures. Combining this finding with the finding that intense deliberate practice alone did not reduce gaming tendencies (Sect. 5.1), one promising direction is to introduce motivational interventions or designs that could maintain or promote perceived competence or self-efficacy in the task domain under intense deliberate practice, to reach a potential multiplier effect of both cognitive and motivational interventions.

## Footnotes

1 In some works, the avoidance of learning has not been explicitly stated in the definition of gaming the system, but they have provided analyses that showed that detected gaming was associated with less learning (Baker et al. 2004a, b; Walonoski and Heffernan 2006a), which converges to the same conclusion here.

2 In the current paper, *gamed* or *gaming* corresponds to *detected* gaming by a gaming detector, which may or may not include productive behaviors. Readers need to interpret such words in context.

3 This KC model was constructed using the new data collected from the experiment (including new tasks in the redesigned tutor), which was different from the KC models constructed before the experiment based on older data and used in the running tutors.

4 The positive (rather than the expected negative) correlation rho values in some cells under the 19F column in Table 11 are considered as a result of statistical noise, since none of these values are even marginally significant.

5 Treating a categorical variable with few levels as a random factor may lead to imprecise estimates (Bolker 2022). Thus, we do not consider this as a next step when the number of formats is small (e.g., < 10).
